# A Unique Four-Hub Protein Cluster Associates to Glioblastoma Progression

**DOI:** 10.1371/journal.pone.0103030

**Published:** 2014-07-22

**Authors:** Pasquale Simeone, Marco Trerotola, Andrea Urbanella, Rossano Lattanzio, Domenico Ciavardelli, Fabrizio Di Giuseppe, Enrica Eleuterio, Marilisa Sulpizio, Vincenzo Eusebi, Annalisa Pession, Mauro Piantelli, Saverio Alberti

**Affiliations:** 1 Unit of Cancer Pathology, Ce.S.I., Foundation University “G. d'Annunzio,” Chieti, Italy; 2 School of Human and Social Science, University “Kore” of Enna, Enna, Italy; 3 Molecular Neurology Unit, Ce.S.I., University “G. d'Annunzio,” Chieti, Italy; 4 Aging Research Center, Ce.S.I., University “G. d'Annunzio” Foundation, Chieti, Italy; 5 Department of Experimental and Clinical Sciences, School of Medicine and Health Science, University “G. d'Annunzio,” Chieti, Italy; 6 StemTeCh Group, Chieti, Italy; 7 Department of “Tutela Salute Donna, Vita nascente, Bambino e Adolescente,” Catholic University of the Sacred Heart, Policlinico Universitario “Agostino Gemelli,” Roma, Italy; 8 Section of Surgical Pathology, “M. Malpighi,” Bellaria Hospital, Bologna, Italy; 9 Department of Neuroscience, Imaging and Clinical Sciences, University “G. d'Annunzio,” Chieti, Italy; Beijing Tiantan Hospital, Capital Medical University, China

## Abstract

Gliomas are the most frequent brain tumors. Among them, glioblastomas are malignant and largely resistant to available treatments. Histopathology is the gold standard for classification and grading of brain tumors. However, brain tumor heterogeneity is remarkable and histopathology procedures for glioma classification remain unsatisfactory for predicting disease course as well as response to treatment. Proteins that tightly associate with cancer differentiation and progression, can bear important prognostic information. Here, we describe the identification of protein clusters differentially expressed in high-grade versus low-grade gliomas. Tissue samples from 25 high-grade tumors, 10 low-grade tumors and 5 normal brain cortices were analyzed by 2D-PAGE and proteomic profiling by mass spectrometry. This led to identify 48 differentially expressed protein markers between tumors and normal samples. Protein clustering by multivariate analyses (PCA and PLS-DA) provided discrimination between pathological samples to an unprecedented extent, and revealed a unique network of deranged proteins. We discovered a novel glioblastoma control module centered on four major network hubs: Huntingtin, HNF4α, c-Myc and 14-3-3ζ. Immunohistochemistry, western blotting and unbiased proteome-wide meta-analysis revealed altered expression of this glioblastoma control module in human glioma samples as compared with normal controls. Moreover, the four-hub network was found to cross-talk with both p53 and EGFR pathways. In summary, the findings of this study indicate the existence of a unifying signaling module controlling glioblastoma pathogenesis and malignant progression, and suggest novel targets for development of diagnostic and therapeutic procedures.

## Introduction

In 2013, more than 23,000 individuals were expected to be diagnosed with primary tumors of brain and central nervous system and more than 14,000 deaths were expected in the US alone [1]. The World Health Organization defines pilocytic (Grade I) and diffuse (Grade II) astrocytomas as low-grade brain tumors; anaplastic astrocytomas (Grade III) and glioblastomas (Grade IV; also designated as glioblastoma multiforme, GBM) are high-grade malignant tumors [2],[3]. With an annual incidence of 2–3 per 100,000 in Europe and US, GBM is the most frequent and aggressive form of brain tumor (60–70% of total malignant gliomas), and is essentially incurable [3], [4]. GBM consists of poorly differentiated, highly invasive neoplastic astrocytes; histopathological features include cellular polymorphism, nuclear atypia, mitotic activity, vascular thrombosis, microvascular proliferation and necrosis [5]. Regional heterogeneity of GBM frequently causes diagnostic discrepancies (≥20% of cases). Moreover, a high percentage of gliomas, such as mixed oligoastrocytomas and lower-grade gliomas, remain difficult to categorize reproducibly due to considerable histological overlap. These factors can compromise choice as well as effectiveness of therapeutic options [6]. Histopathologic diagnosis can be further compromised when only small biopsies are available. Additional molecular markers are thus urgently needed to efficiently discriminate among patients with distinct outcomes.

Loss of *PTEN*, amplification of *EGFR* and alterations of *TP53*, *PDGFRA* and *CDKN2A/P16* are frequently found to be associated with GBM pathogenesis [5], [7]. Primary GBMs develop *de novo* after a short clinical history and without evidence of precursor lesions, whereas “secondary” GBMs arise from pre-existing diffuse or anaplastic astrocytomas. The signaling pathways responsible for development and growth of primary versus secondary GBM appeared as profoundly diverse, suggesting these two types of GBM to be different disease entities. Rather diverse genetic signatures were further proposed in the attempt of explaining GBM pathogenesis and heterogeneity [5], [8]–[12]. However, the actual impact of genetic signatures for GBM diagnosis and prognosis remains to be defined.

Genomic and transcriptomic data have provided key insight in GBM pathophysiology [11]–[14]. Corresponding insight into GBM proteomics [15] has not yet been achieved. Proteomic analysis of low- and high-grade tumors has tried to fill this gap [16]–[20], but analysis of specific protein markers has largely failed to provide a comprehensive view of GBM pathology.

In this study, we set to identify significantly modulated protein clusters that may bear functional impact and robustly explain distinct, relevant GBM pathology components. Proteomic analysis of human high-grade tumors, low-grade tumors and control tissue samples from normal brain cortex was thus systematically intersected through multivariate statistical procedures (principal component analysis, PCA and partial least square-discriminant analysis, PLS-DA). Using this approach, we were able to identify protein clusters discriminating tumors from normal tissues as well as high-grade from low-grade gliomas. Connectivity network analysis then allowed to discover a GBM control module that encompassed four major signaling hubs centered on Huntingtin, HNF4α, 14-3-3ζ and c-Myc. This proteomic signature was shown to underlie p53 and EGFR signaling, as an interconnected network. The GBM control module is candidate to be used as diagnostic biomarker and as target for therapeutic intervention. It may also help drafting a unifying model for glioblastoma appearance.

## Materials and Methods

### Patients and tissue specimens

Bioptic samples from low-grade and high-grade glioma patients were frozen in liquid nitrogen and stored at −80°C at the Section of Pathology “M. Malpighi” of the Bellaria Hospital, University of Bologna, between 1990 and 2002. Corresponding formalin-fixed paraffin embedded (FFPE) samples were stained with haematoxylin-eosin for routine histological diagnosis. The protocol of this study was approved by the board of the Ministry of the University and Research (“Novel technologies for glioblastoma assessment”, FISR Neurobiotechnologies, Grant N 481). Informed consent was previously obtained as indicated in Marucci et al. [21].

A total of 10 low-grade glial tumors (4 oligodendrogliomas, OL, 4 pilocytic astrocytomas, PA and 2 fibrillary astrocytoma, FA) and 25 GBMs were collected. All samples were re-staged and graded by expert pathologists according to 2007 WHO central nervous system tumor classification [2]. Control samples were tissues from five normal cortices from different brain regions, as obtained at autopsy from individuals deceased from diseases not involving the brain.

### Reagents and chemicals

All reagents and chemicals are purchased from Sigma-Adrich (St. Louis, MO, USA), Bio-Rad Laboratories (Hercules, CA, USA) and GE Healthcare (Little Chalfont, UK).

### Brain tumor lysates

Frozen brain tumor specimens were thawed on ice and resuspended in 2-DE lysis buffer (8 M urea, 40 mM Tris base, 65 mM DTT). Tumor lysates were briefly sonicated in Eppendorf (Hamburg, Germany) tubes with three 10-sec bursts, in 4% CHAPS. The lysates were centrifuged for 15 min at 12000 rpm to remove cell debris. Lysate supernatants were then processed for 2D PAGE analysis (Two dimensional polyacrylamide gel electrophoresis).

### 2D Electrophoresis

The first dimension was run over non-linear immobilized pH gradients (3.5–10.0 NL IPG 18 cm) (Pharmacia-Hoeffer Biotechnology AB, CA, USA). Hydration was achieved overnight in the reswelling cassette with 25 ml of a solution containing 8 M urea, 2% CHAPS (w/v), 10 mM DTE, 2% (v/v) pH 3.5–10 Ampholites, bromophenol blue and 200 µg of protein extract [22]. Run strips were equilibrated in 50 mM Tris-HCl pH 8.4, 6 M urea, 2% (w/v) DTE, 2% (w/v) SDS, 30% (v/v) glycerol for 12 min. Sulphydrilic groups were blocked in 2.5% (w/v) iodoacetamide, 50 mM Tris-HCl pH 6.8, 6 M urea, 2% (w/v) SDS, 30% (v/v) glycerol, bromophenol blue for 5 min.

The SDS–PAGE (Sodium dodecyl sulfate polyacrylamide gel electrophoresis) dimension was run in a vertical gradient acrylamide/PDA (9–16% T/2.6% C) slab gel. Sodium thiosulfate was used as an additive to reduce background in the silver staining. A constant current of 40 mA/gel was applied [23]. Gels were removed from glass plates, washed in deionized water for 5 minutes, and stained with ammoniacal silver as described by [22]. Preparative gels were stained with the Protea silver stain kit compatible with mass spectrometry analysis (Protea Bioscience, Morgantown, WV, USA).

### Image analysis

The GS-700 Densitometer Gel Doc (Bio-Rad Laboratories, Hercules, CA, USA) was used as scanning device. Protein spots were detected using ImageMaster 2-D Platinum software, version 6.0 (GE Healthcare, Little Chalfont, UK). Spot borders were visually inspected and misidentification caused by confluent spots, artifacts and low signal to noise ratio, were manually corrected. Parameters like “saliency” (a measure of spot curvature) and “min area” (lowest area threshold under which spots are considered artefacts) were used to identify ‘true’ and ‘false’ protein spots. Manual contour drawing was then applied in all cases of sub-optimal spot auto-detection. This procedure was validated by assessing total spot numbers and spot volume ratios before and after background subtraction.

In order to optimize quantitative analysis of protein spots, the volume of each candidate spot was normalized using 4 surrounding landmark spots localized in areas closeby to the spot of interest, i.e. within corresponding background grey and with analogous signal staining exposure. Landmarks ratios were used for first-level normalization. Sums of landmark signals were then used for target spot normalized quantification. Using these criteria, spot detection and quantification were obtained, which minimized intensity variations among the gels, as assessed by Mann-Whitney test (p<0.05). Spots with more than 50% of data missing were not included in subsequent analytical steps.

Expression data of each identified spot were plotted into a frequency histogram to highlight main differences between analyzed sets, to visualize subtypes within the same histopathological group and to assess valued distribution shapes. The Shapiro-Wilk test was utilized to assess for normal (Gaussian) distributions.

### Mass spectrometry analysis

After tryptic in gel-digestion, overnight at 37°C [24], the differentially expressed spots were excised from preparative gels and analyzed by mass spectrometry (MS) to identify amino acid sequences, using a Bruker Ultraflex III (Bruker, Bremen, Germany) operating in reflectron mode. This instrument was equipped with a Nd:YAG smartbeam laser to acquire positive-ion MALDI mass spectra over a mass range of m/z 800–4000. Spectral processing and peak list generation were implemented by Bruker flexAnalysis software (version 3.3, Bruker Daltonics) for MS and MS/MS spectra. For each protein spot, the most intense precursor ion signals in each MS spectrum were analyzed by MS/MS fragmentation in LIFT mode. α-cyano-4-hydroxycinnamic acid was used as matrix. Spot identifications were performed by querying the Mascot database. Trypsin cut, carbamidomethyl (C) as fixed, oxidation (M) as variable, a maximum of one missed cleavage allowed, were imposed as modifications in the search parameters. Peptide tolerance and MS/MS tolerance were set at 250 ppm and 0.5 Da respectively.

### Univariate and multivariate statistical analysis

Univariate statistical analyses were performed with GraphPad Prism (GraphPad Software Inc., La Jolla, Ca) and XLStat (Addinsoft, Paris, France). Spearman's correlation analysis was performed using MetaboAnalyst 2.0 software (www.metaboanalyst.ca) [25]–[27]. Multivariate statistical analysis and data modeling were performed using MetaboAnalyst 2.0 (www.metaboanalyst.ca) [25]–[27] and SIMCA 13 (Umetrics, Umea, Sweden) [28] software packages.

Column-wise normalization was applied to provide Gaussian-like distributions [25], [29]. Analyses were then performed on autoscaled data (mean-centered and divided by the standard deviation of each variable) [30]. A diagnostic plot was utilized to represent normalization procedures for normal distribution assessments [29]. As examples, value intensities for e.g. APOA1, PRDX2, ALDOC, CRYAB_b, TTHY are ≥3- fold higher than others (e.g. NDUS1, QCR1, NFM, ACTB), thus inducing a skewed distribution. After autoscaling normalization, box plots have nearly same mean, standard deviation, and their distribution better matches a Gaussian curve (Figure S1B right) (Kernel density plot right-bottom) [31]. PCA was used as an unsupervised method in order to find the directions of maximum covariance among our protein spots without referring to class labels (tissue samples). This allowed to visualize differences among samples, to detect clustering and pick-up outliers.

PCA condenses datasets to obtain optimal dimensions that best capture signal covariance. However, it fails providing working hypotheses for some causal relations among data subsets [32]. Hence, we went on performing histopathology classification-guided PLS-DA. As many supervised classification algorithms tend to overfit the data [26], [33], PLS-DA model validation was performed as previously described [34]. Briefly, to define the optimal number of PCs (principal components), “7-fold cross-validation” (CV) was applied [35], [36]. Using CV, the predictive power of the model was verified. Two parameters were calculated for evaluating the models: R^2^ (goodness of fit) and Q^2^ (goodness of prediction). A model with Q^2^>0.5 was considered good, Q^2^>0.9 excellent [37], [38]. As cross-validation only assesses the predictive power without a statistical validation, the performance of PLS-DA models was also validated by a permutation test (200 times).

To help interpreting results from PLS-DA, we considered the variable importance in the projection scores (VIP score) and regression coefficients (CoeffCS). This allowed to evaluate protein influence (including prediction performance) on the model and identify the best descriptors of the differences among the three groups. The VIP score is a weighted sum of squares of the PLS loading weights taking into account the amount of explained Y-variation in each dimension [25], [26], [39]. Since the average of squared VIP scores equals 1, the “greater than 1” rule is generally used as a criterion to identify the most significant variables [37], [39]. PLS-DA CoeffCS express the relation between the Y variables (classes) and all the terms in the model and are used for interpreting the influence of the X variables (proteins) on Y. VIP and CoeffCS values are cumulatively calculated from all extracted PLS components. The coefficients express how strongly the Y groups are correlated to the systematic part of each X variable considering all three components.

### Gene ontology, networks and functional analyses

Gene Ontology (GO) analysis was performed using PANTHER 7.2 software (www.pantherdb.org/). The signaling hubs and connectivity networks were obtained using Ingenuity Pathway analysis (IPA, Ingenuity Systems, www.ingenuity.com) and STRING 9.1 (string-db.org) package.

### Western blotting

Expression levels of randomly selected proteins were analyzed by Western blotting in order to validate the dataset identified by proteomic analysis. Blots were incubated with the following primary antibodies (Santa Cruz, Santa Cruz, CA): LDH-B (Q-21) sc-133731 rabbit polyclonal; SOD-1 (V-17) sc-34015 goat polyclonal; APOA-I (FL-267) sc-30089 rabbit polyclonal; Aldolase C Antibody (N-14) sc-12065 goat polyclonal and PRXII (9A1) sc-59660 mouse monoclonal. Signal intensities of the bands were quantified with Image JA 1.46b, using a Kodak grey-scale standards power curve (www.kodak.com) as reference. Band intensity values were normalized versus red Ponceau signals of transferred proteins on Western blot filters. Normalized densitometry values between proteomic gel spots and Western blot bands were correlated with the Spearman's rank correlation analysis, to obtain rho coefficients and corresponding p values.

The expression profiles of hub proteins (Huntingtin, HNF4α, c-Myc, 14-3-3ζ) were also determined on glioma tissue lysates.

### Immunohistochemistry staining

Five µm sections from FFPE samples were stained overnight using antibodies to Huntingtin (Millipore MAB2166; mouse monoclonal, clone 1HU-4C8, 1∶150), HNF4α (AbCam ab41898; mouse monoclonal, clone K9218, 1∶70), 14-3-3ζ (AbCam ab51129; rabbit polyclonal, 1∶50) and c-Myc (AbCam ab32072; rabbit monoclonal, clone Y69, 1∶100). Antigen retrieval was performed using hot citrate buffer pH 6.0 (Huntingtin, HNF4α, and 14-3-3ζ) or 1 mmol/L EDTA pH 8.0 (c-Myc). Antigen-antibody reactions were visualized using a polymer-based detection system (EnVision Kit, Dako), using diaminobenzidine as chromogen.

### Immunohistochemistry data bank meta-analysis

Publicly available databases [40], [41], containing high-resolution IHC (Immunohistochemistry) images extending proteome-wide were analyzed for patterns of expression of GBM-driving engines. The Human Protein Atlas (v. 12, www.proteinatlas.org) provides spatial distribution and expression data from 16621 proteins/21984 antibodies in different normal human tissues and different cancer types. The expression profiles of hub proteins were generated for antibody staining parameters, intensity, and fraction of positive cells in control versus glioma arrays. EGFR and p53 IHC staining were used as internal benchmark for performance assessment and quantification standards.

## Results

### Proteomic analysis

**Figure 1:**
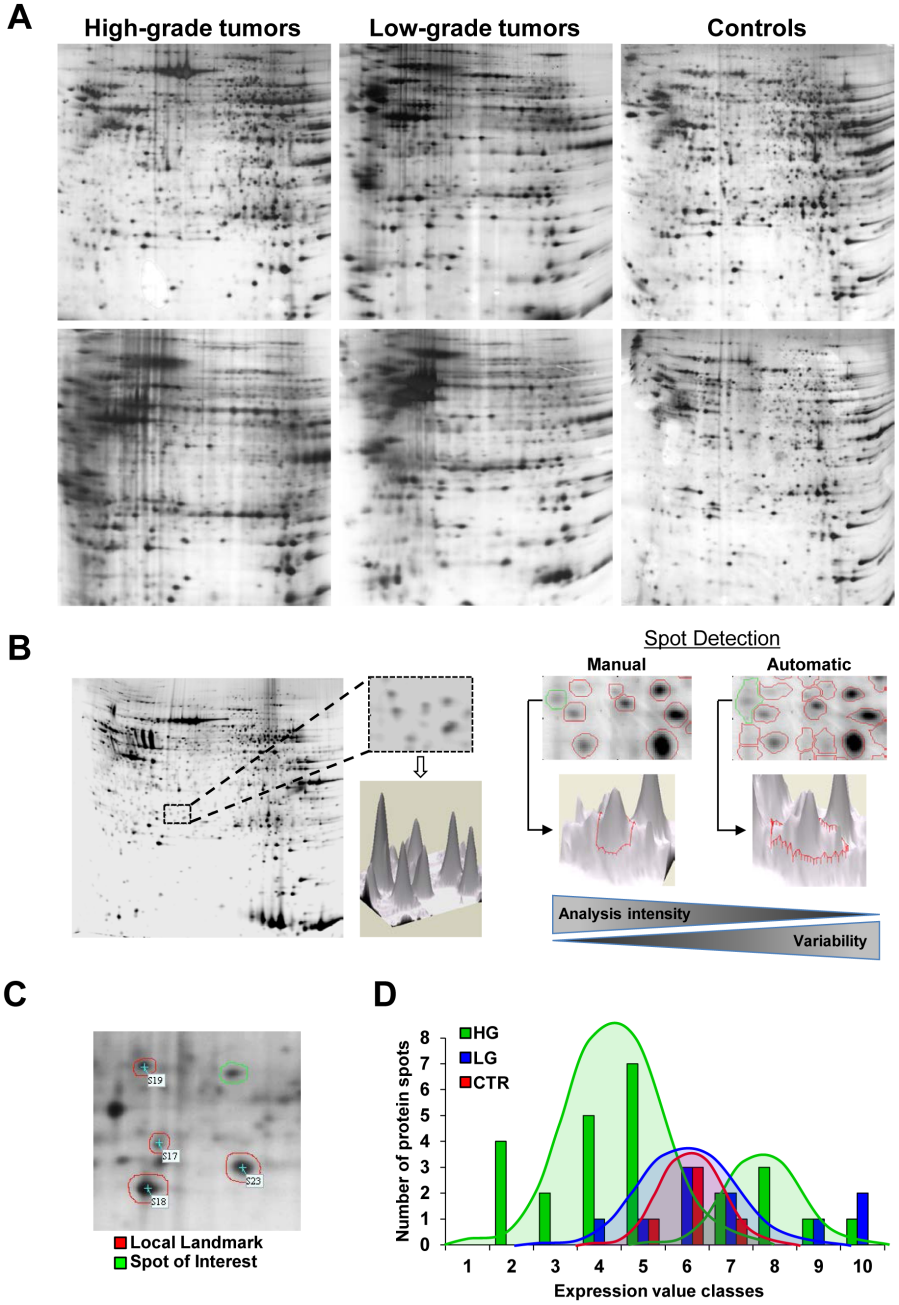
Gel analysis, spot detection and edge improvement. (A) Representative 2-DE gels from normal brain, low-grade and high-grade gliomas. Proteins from normal and tumor brain tissues were processed as described in materials and methods. Protein spots were visualized by ammoniacal silver staining. (B) 3-D representation of protein volume spots and comparison between automatic and manual procedures for definition of protein spots and edges. Manual strategy is time-consuming but allows reducing the number of misidentifications and improving the quantification of the same protein markers between different gels. (C) Normalization of density values of differentially expressed spots (green) by using surrounding landmarks (red). (D) High-grade histotype tumors (green) showing a bimodal distribution of triosephosphate isomerase (TPIS1) expression values. In low-grade tumors (blue) and control samples (red) the values appear to follow a typical Gaussian (normal) distribution.

Twenty-five high-grade GBMs, 10 low-grade gliomas and 5 tissue samples from normal brain cortex were analyzed by 2D PAGE (**Figure 1A**). Clinical data from brain tumor patients are summarized in **Table 1** [21].

**Table 1:** Clinical data from tumor patients.

ID	Age	Site	Histology	OS (m)
**High-grade**				
GB1	68	Fr	*de novo*	0.5 (OD)
GB3.1	69	Te	*de novo*	4.9
GB3.2	69	Te	*de novo*	4.9
GB6	71	Pa	*de novo*	14.2
GB9	33	Te	*de novo*	18.6
GB10	67	Te	*de novo*	1.6
GB12	73	Pa	*de novo*	1.9
GB13	67	Pa	*de novo*	10.4
GB15	59	Te	*de novo*	9.1
GB18	53	Fr	*de novo*	26.3
GB19	59	Fr-Te	*de novo*	11.5
GB20	57	Fr	*de novo*	13.2
GB26	34	Fr	secondary	10.1
GB28	50	Fr	*de novo*	N/A
GB29	58	Fr	*de novo*	N/A
GB31	60	Fr	*de novo*	N/A
GB33	56	Fr	*de novo*	N/A
GB34	36	Fr	*de novo*	N/A
GB35	50	Fr	*de novo*	14.2
GB36	69	Te	*de novo*	1.0
GB39	67	Pa	*de novo*	7.4
GB40	64	Te	*de novo*	9.9
GB45	69	Te-Pa	*de novo*	13.2
GB46	79	Fr	*de novo*	alive
GB47	73	Pa	*de novo*	36.0
**Low-grade**				
FA24	29	Pa	*de novo*	alive
FA34	34	Pa	*de novo*	134
OL29	32	Fr	*de novo*	105
OL30	60	Suprat	*de novo*	alive
OL31	60	Fr	*de novo*	alive
OL32	3	Fr	*de novo*	alive
PA21	7	Cereb	*de novo*	alive
PA22	20	Cereb	*de novo*	alive
PA28	26	Te	*de novo*	alive
PA33	41	Fr	secondary	132

ID: sample code number; OS (m): overall survival (months); GB: glioblastoma mutiforme; OL: oligodendroglioma; FA: fibrillary astrocytoma; PA: pilocytic astrocytoma; Fr: frontal lobe; Te: temporal lobe; Pa: parietal lobe; Suprat: supratentorial; Cereb: cerebellum; OD: dead from other disease.

To improve quantification accuracy and allow robust statistical analysis of 2D gel data [42], we optimized image processing, spot detection and signal quantification procedures by applying operator-guided background subtraction and spot contour optimization (**Figure 1B** **and S1A**). These procedures allowed to obtain 49±15% increase of the detected signal (spot volume upon spot contour optimization) (**Table S1**), and better spot detection (+16±2%), as compared with the basic/automatic procedure. Most spot variation was not detectable across all samples; therefore, protein spots were marked as “differential” if expression changes could be observed in at least one tumor sample versus all control samples. The statistical distributions were found to be not-normal and often bimodal for most protein spots (**Figure 1D**). Forty-eight protein markers were identified by MS (**Table 2** **, Figure S2, Supplemental Material S1**), and their expression levels were quantified upon the image optimization procedures described above (**Figure S3A and Table S2A,B**). For enhancing quantification robustness, the density value of each candidate spot was normalized versus 4 surrounding landmarks (**Figure 1C**, **Table S2A**) as described in Materials and Methods section. This allowed to obtain robust quantification, through compensating for local staining dishomogeneities.

**Table 2:** Proteins identified by MALDI-TOF/TOF.

Protein name^a^	Accession number^b^	Uniprot name^c^	Gene name^d^	Mascot protein score^e^	Total Ion score^f^	Protein coverage %^g^	Peptides number^h^	Experimental MW (kD)/IP^i^
**14 kDa phosphohistidine phosphatase**	**Q9NRX4**	**PHP14**	**PHPT1**	79	79	18	2	14.5/5.4
**2′,3′-cyclic-nucleotide 3′-phosphodiesterase**	**P09543**	**CN37**	**CNP**	75	77	5	3	42.3/9.8
**26S proteasome non-ATPase regulatory subunit 13**	**Q9UNM6**	**PSD13**	**PSMD13**	82	81	10	3	39.4/5.6
**3-hydroxyacyl-CoA dehydrogenase type-2**	**Q99714**	**HCD2**	**HSD17B10**	335	225	34	7	24.9/7.5
**6-phosphogluconolactonase**	**O95336**	**6PGL**	**PGLS**	461	461	36	7	27.8/5.7
**Actin, cytoplasmic 1**	**P60709**	**ACTB**	**ACTB**	168	101	19	6	48.0/5.7
**Alpha-centractin**	**P61163**	**ACTZ**	**ACTR1A**	240	240	15	4	43.8/6.2
**Alpha-crystallin B chain (b)**	**P02511**	**CRYAB**	**CRYAB**	146	145	14	2	21.4/7.0
**Alpha-crystallin B chain (a)**	**P02511**	**CRYAB**	**CRYAB**	173	119	29	5	19.4/6.3
**Apolipoprotein A-I**	**P02647**	**APOA1**	**APOA1**	161	83	27	7	24.5/5.2
**Astrocytic phosphoprotein PEA-15**	**Q15121**	**PEA15**	**PEA15**	225	226	37	4	15.1/4.8
**ATP synthase subunit d, mitochondrial**	**O75947**	**ATP5H**	**ATP5H**	105	52	34	4	22.0/5.1
**Beta-centractin**	**P42025**	**ACTY**	**ACTR1B**	62	62	9	2	43.2/6.0
**Chloride intracellular channel protein 1**	**O00299**	**CLIC1**	**CLIC1**	141	140	13	3	30.3/5.1
**Creatine kinase B-type**	**P12277**	**KCRB**	**CKB**	107	108	16	4	41.3/5.5
**Cytochrome b-c1 complex subunit 1, mitochondrial**	**P31930**	**QCR1**	**UQCRC1**	109	109	16	4	46.3/5.5
**Destrin**	**P60981**	**DEST**	**DSTN**	73	62	7	1	27.6/7.3
**Dihydropyrimidinase-related protein 2**	**Q16555**	**DPYL2**	**DPYSL2**	332	265	20	7	67.0/5.8
**Dimethylarginine dimethylaminohydrolase 1**	**O94760**	**DDAH1**	**DDAH1**	112	112	14	3	37.6/5.5
**F-actin-capping protein subunit alpha-1**	**P52907**	**CAZA1**	**CAPZA1**	78	78	19	3	36.6/5.4
**Fructose-bisphosphate aldolase C**	**P09972**	**ALDOC**	**ALDOC**	464	465	24	6	39.8/6.5
**Glial fibrillary acidic protein**	**P14136**	**GFAP**	**GFAP**	72	72	6	2	24.2/5.0
**GTP-binding nuclear protein Ran**	**P62826**	**RAN**	**RAN**	66	54	6	1	25.5/7.93
**Hemoglobin subunit alpha**	**P69905**	**HBA**	**HBA1/2**	298	235	28	3	28.9/8.8
**Hemoglobin subunit delta**	**P02042**	**HBD**	**HBD**	339	244	62	7	28.3/8.9
**Inorganic pyrophosphatase**	**Q15181**	**IPYR**	**PPA1**	74	74	9	2	33.9/5.5
**Inositol monophosphatase 1**	**P29218**	**IMPA1**	**IMPA1**	114	114	11	3	28.9/5.0
**Isocitrate dehydrogenase [NAD] subunit alpha, mitochondrial**	**P50213**	**IDH3A**	**IDH3A**	187	188	11	4	38.1/5.7
**L-lactate dehydrogenase B chain**	**P07195**	**LDHB**	**LDHB**	514	515	30	7	36.9/5.7
**Malate dehydrogenase cytoplasmic**	**P40925**	**MDHC**	**MDH1**	288	288	14	4	37.1/6.5
**NADH-ubiquinone oxidoreductase 75 kDa subunit, mitochondrial**	**P28331**	**NDUS1**	**NDUFS1**	153	108	12	6	87.3/5.7
**Neurofilament medium polypeptide**	**P07197**	**NFM**	**NEFM**	245	156	18	11	54.3/5.6
**Nucleoside diphosphate kinase A**	**P15531**	**NDKA**	**NME1**	291	215	44	5	18.5/5.7
**Peroxiredoxin-2**	**P32119**	**PRDX2**	**PRDX2**	385	385	15	3	22.2/5.5
**Peroxiredoxin-3 (a)**	**P30048**	**PRDX3**	**PRDX3**	84	84	10	2	23.9/5.7
**Peroxiredoxin-3 (b)**	**P30048**	**PRDX3**	**PRDX3**	162	140	10	2	23.5/6.0
**Peroxiredoxin-5**	**P30044**	**PRDX5**	**PRDX5**	386	326	43	4	16.0/7.6
**Phosphatidylethanolamine-binding protein 1/Raf kinase inhibitor protein (RKIP)**	**P30086**	**PEBP1**	**PEBP1**	554	554	43	5	22.3/8.0
**S-formylglutathione hydrolase**	**P10768**	**ESTD**	**ESD**	53	42	8	1	32.0/6.4
**SH3 domain binding glutamic acid-rich like protein**	**O75368**	**SH3L1**	**SH3BGRL**	63	63	9	1	14.4/5.2
**Stathmin**	**P16949**	**STMN1**	**STMN1**	85	85	17	2	17.1/5.7
**Superoxide dismutase [Cu-Zn]**	**P00441**	**SODC**	**SOD1**	130	130	17	2	17.8/5.7
**Transketolase**	**P29401**	**TKT**	**TKT**	211	210	10	4	68.2/8.8
**Transthyretin**	**P02766**	**TTHY**	**TTR**	105	87	9	1	15.6/5.5
**Triosephosphate isomerase**	**P60174**	**TPIS**	**TPI1**	546	418	31	8	26.6/6.1
**Tubulin alpha-1B chain**	**P68363**	**TBA1B**	**TUBA1B**	272	274	17	5	37.6/5.4
**Ubiquitin carboxyl-terminal hydrolase isozyme L1**	**P09936**	**UCHL1**	**UCHL1**	64	54	8	1	25.6/5.2
**Ubiquitin-conjugating enzyme E2 N**	**P61088**	**UBE2N**	**UBE2N**	182	172	36	4	15.7/5.8
**Vesicle-fusing ATPase**	**P46459**	**NSF**	**NSF**	114	113	6	4	78.0/6.4
**Zinc finger protein 555**	**Q8NEP9**	**ZNF555**	**ZNF555**	57	ND	12	6	18.7/7.6

a : protein name;

b : UniProt accession number;

c : UniProt identification name;

d : Gene name;

e : Mascot/protein score;

f : Total Ion Score.

g : % Protein coverage and ^h^: Peptides number are the percent fraction of the total protein length and number of peptides identified by MS spectrometry analysis, respectively.

i : MW (kD)/IP, experimental molecular weight and isoelectric point.

**Figure 2:**
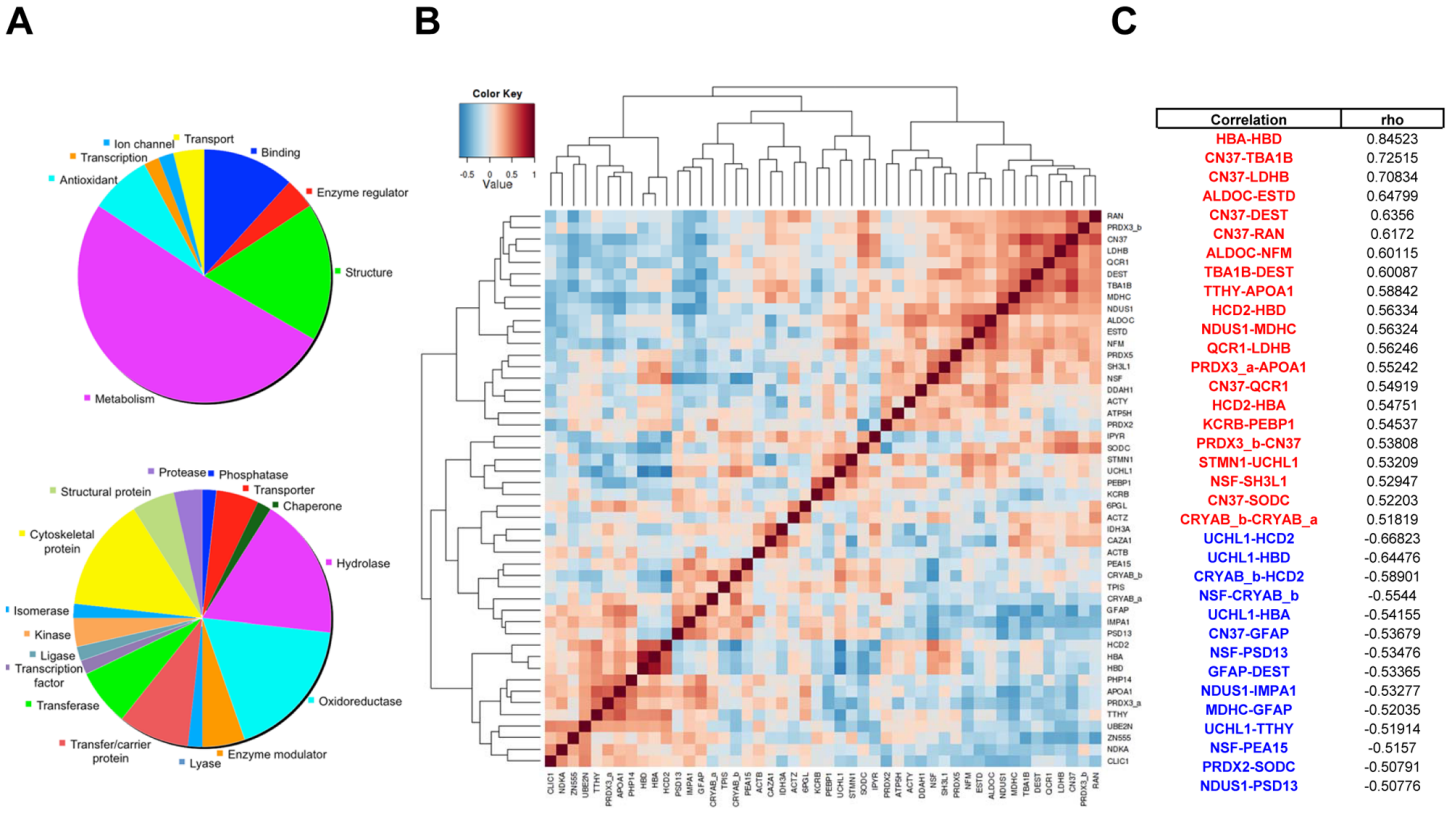
Classification of identified spots and correlation analysis. (A) GO pie charts show PANTHER classifications made according to the associated Molecular function (top) and Protein class (bottom). (B) Graphical representation of Spearman's correlation matrix. Heatmap shows Spearman's correlation between differentially expressed protein spots. Each column and row defines an individual variable. Positive correlation values are in red, and negative correlation values are in blue. Hierarchical clustering was applied to both dimensions. (C) Positive (rho ≥0.5) and negative (rho ≤−0.5) correlations are listed in red and blue, respectively.

GO analysis was performed, revealing the largest protein classes to have metabolic (26 proteins), structural (9) molecule binding (6) and antioxidant (4) activities (**Figure 2A** **, top**). Dual/multiple activities accounted for redundant/over-represented functions. Most detected proteins were hydrolases (10), oxidoreductases (10), components/interactors of the cytoskeleton (8), transfer/carrier proteins (5) and transferases (4). (**Figure 2A** **, bottom**).

### Spearman's correlation analysis

Negative or positive correlations were globally revealed by Spearman's correlation analysis (**Figure 2B**). Highest positive correlations (**Figure 2C** **, red**) were found to occur between HBA and HBD; CN37 and TBA1B; CN37 and LDHB; ALDOC and ESTD; CN37 and DEST; CN37 and RAN; ALDOC and NFM; TBA1B and DEST. Highest negative correlations (**Figure 2C** **, blue**) were observed between UCHL1 and HBD, and between UCHL1 and HCD2 (**Table S3**).

### PCA analysis

The proteomic matrix was processed by scaling protein expression values in order to reduce potential systematic bias and make the variables comparable in magnitude to each other [25], [27], as indicated. The data scaling results and normalization procedures, are summarized graphically in the Figure S1B. The horizontal box plots represent the distributions of individual variables, the bottom curves show the global data distribution based on kernel density estimation (Figure S1B).

**Figure 3:**
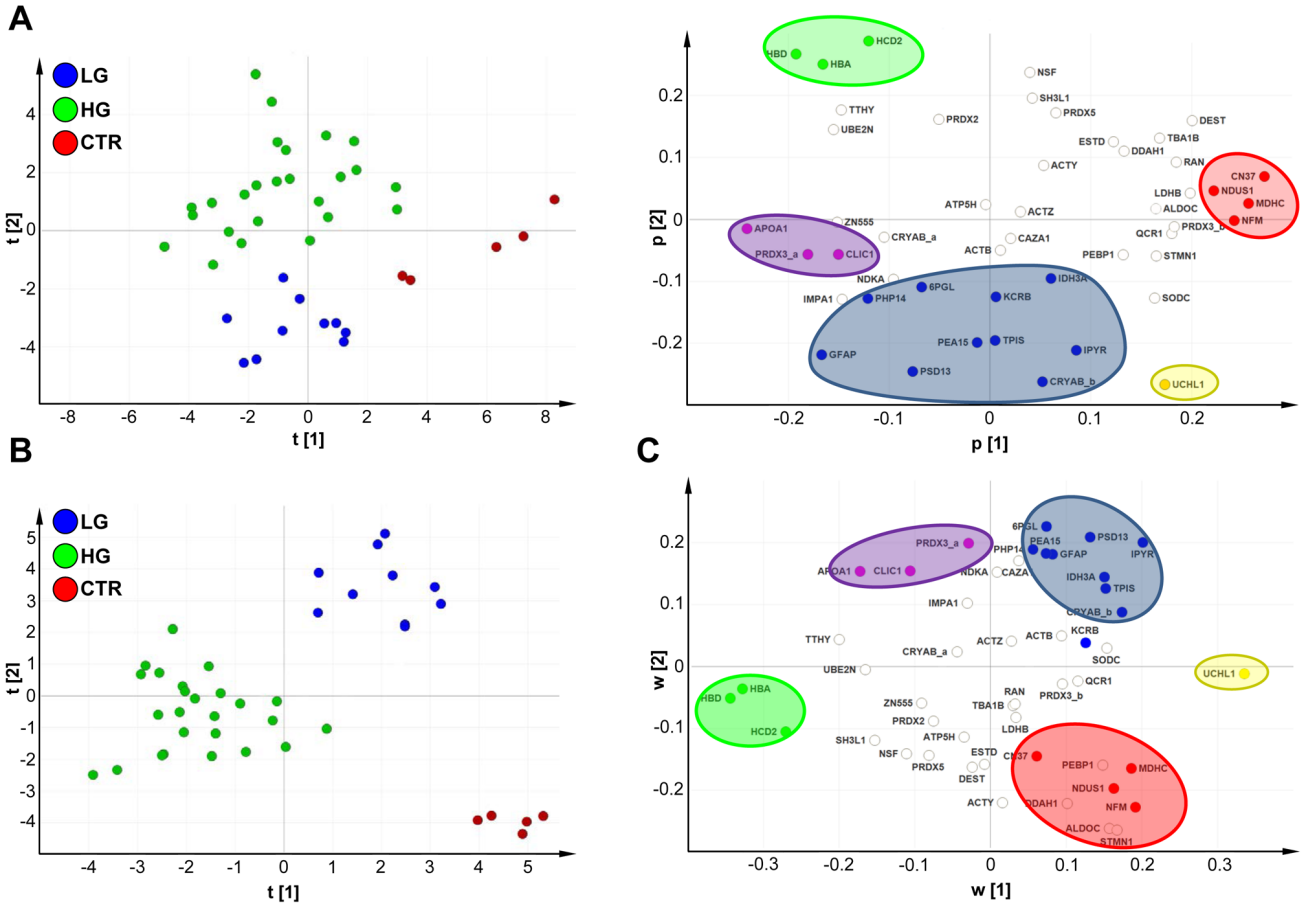
PCA and PLS-DA models. (A, left) PCA score plot showing separation between control samples, low-grade and high-grade tumors. (A, right) PCA loading plot showing the proteins (variables) responsible for discrimination between the groups. (B) PLS-DA score plot showing separation between control samples, low-grade and high-grade tumors. (C) Overlapping of PCA correlation groups (as shown in Figure 3A, right panel) to the PLS-DA weight plot in order to enhance the discriminating power of identified markers. Colored ovals and solid circles represent PLS-DA and PCA protein clusters, respectively. Color code: red, controls; blue, low-grade tumors; green, high-grade tumors; magenta, low and high-grade tumors; yellow, low-grade tumors and controls.

Then, we went on to utilize PCA as an unsupervised multivariate method for analyzing the dataset and identifying the best discriminators among sample classes. PCA score plots were generated (**Figure 3A**), where each axis represented a PC identifying linear combinations of the most tightly interconnected proteins/signaling networks [32], [43]. Samples with similar protein expression profiles/PC scores clustered together with striking fitness. PC1 (score vector t1) was found to discriminate controls from tumors; PC2 (score vector t2) separated low-grade from high-grade gliomas (**Figure 3A** **, left**). Seven major discriminators between control and tumor samples were found: expression levels of APOA1, PRDX3_a and CLIC1 were higher in tumors than in normal brain cortex, whereas significantly lower levels of NFM, CN37, NDUS1 and MDHC were found in tumors as compared with normal tissues (component PC1, **Figure 3A** **, right**). Thirteen major discriminators between low- and high-grade tumor samples were found: expression levels of HCD2, HBA and HBD were strongly up-regulated in high-grade gliomas, whereas CRYAB_b, IPYR, TPIS, PEA15, PSD13, GFAP, PHP14, 6PGL, KCRB, IDH3A had higher expression in low-grade than high-grade tumors (component PC2, **Figure 3A** **, right**). PCA analysis also revealed that UCHL1 have a high discriminating power of this marker when control/low-grade samples were compared with high-grade tumors. Global sets of protein marker with higher (**Figure S3B,C**) and lower discriminating power (**Figure S3D**) were identified.

### PLS-DA analysis

**Figure 4:**
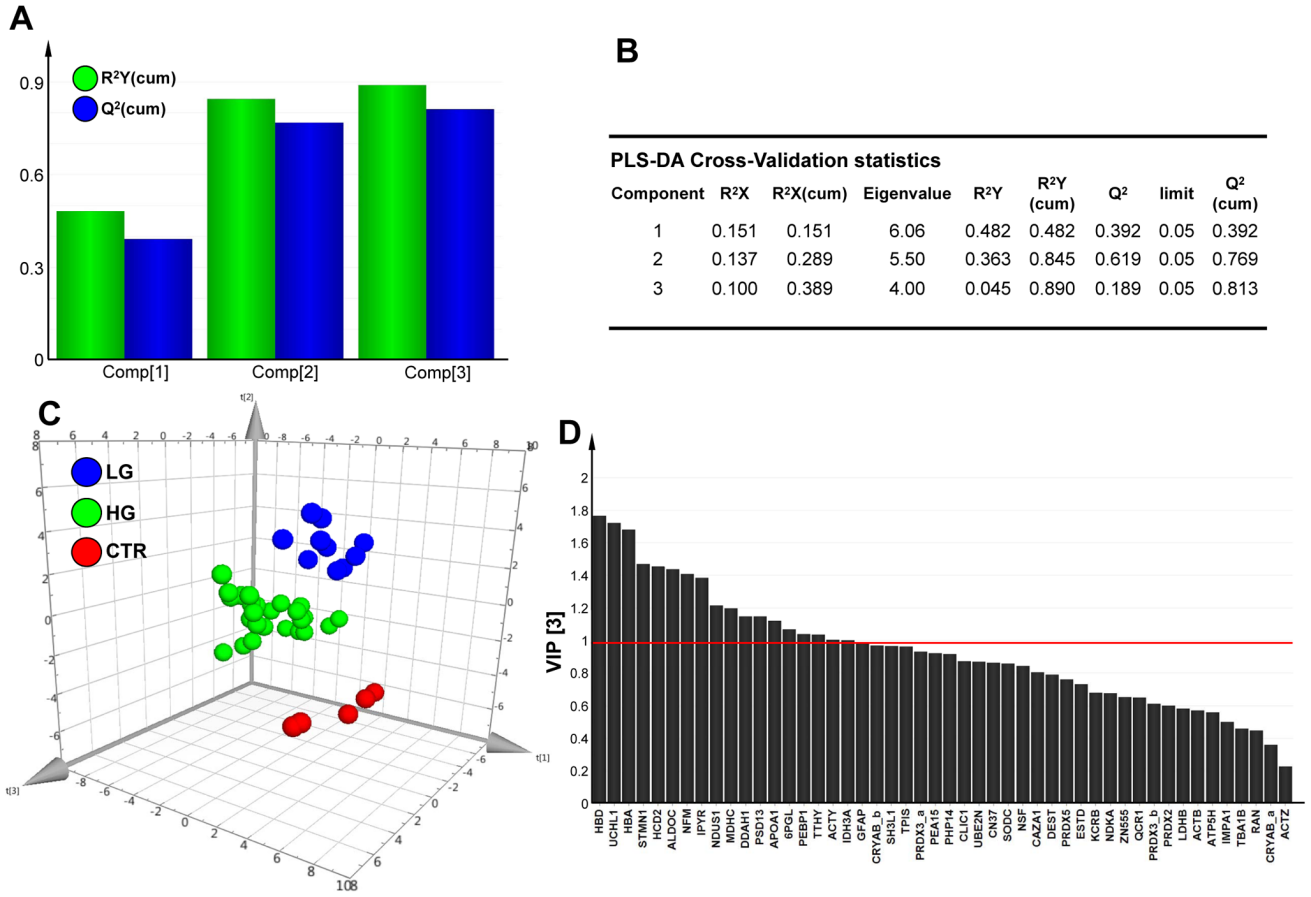
PLS-DA cross-validation, performance and protein VIP scores. (A–B) Bar plot showing the performance measures (R^2^Ycum and Q^2^cum) using different number of components. The selected performance measure Q^2^ shows the three-component model performs as the best one. R^2^X: portion of the variation of X explained by specified PC; R^2^X(cum) Cumulative explained portion of X set variation; Eigenvalue: number of variables (K) times R^2^X; R^2^Y: portion of the Y set variation modeled by the PC; R^2^Y(cum): cumulative modeled variation of Y set; Q^2^: overall cross-validated R^2^ for the specific PC; Limit: threshold cross-validation for the specific PC; Q^2^(cum): cumulative Q^2^ up to the specified component, is a model predictive power according to cross validation. Unlike R^2^X(cum), Q^2^(cum) is not additive. (C) 3-D score plot. The samples are represented in the 3-D score plot, the first three components (PC1, PC2, PC3) are reported accounting 15.1%, 13.7% and 10% of total variation respectively. (D) Proteins able to discriminate between controls, low-grade tumors and high-grade tumors, ordered by VIP score. VIP scores ≥1 are significant (above the red line) and indicate important X variables (proteins) that predict Y responses (tissue samples).

To verify the strength of the unsupervised PCA analysis, and to further build on it, we analyzed the dataset on the basis of known classes (controls vs high-grade tumors vs low-grade tumors) using a supervised PLS-DA method [37], [38], [44], [45]. This model was found to have strong goodness of fit (cumulative R^2^Y = 0.890) and prediction power (cumulative Q^2^ = 0.813) (**Figures 4 A,B**). The separation between normal brain tissues, low-grade and high-grade gliomas yielded a staggering clear discrimination (**Figure 3B**). Most significant separations were explained by a three-component model, where principal components PC1, PC2 and PC3 represented 15.1%, 13.7% and 10% of the total variance in the protein spot-matrix, respectively (**Figure 4**). Permutation tests were carried out in order to validate the PLS-DA model [37]: as shown in **Figure S4A**, the original model was found to have higher R^2^ and Q^2^ values than the permuted models, and negative Q^2^ values were obtained for all three permuted groups tested.

A PLS-DA loading plot was generated in order to find major discriminants between the groups analyzed (**Figures 3C**). Eleven major discriminators between control and tumor samples were identified: APOA1, CLIC1 and PRDX3_a were over-expressed, whereas NFM, CN37, NDUS1, MDHC, ALDOC, STMN1, PEBP1 and DDAH1 were down-regulated in tumors as compared with control samples. Twelve major discriminators between low- and high-grade gliomas were identified: HCD2, HBA and HBD were up-regulated in high-grade gliomas, whereas expression levels of CRYAB_b, IPYR, TPIS, PEA15, PSD13, GFAP, IDH3A, 6PGL and PHP14 were found to be higher in low-grade than in high-grade tumors. The regression coefficients calculated for PLS-DA outcomes confirmed the power of the identified clusters in distinguishing among sample classes (**Figure S4B**). Next, we calculated the VIP score for each protein in our dataset. Out of 48 variables analyzed as potential predictors, the following 18 descriptors were found to significantly contribute to the classification model (VIP score ≥1): HBD, UCHL1, HBA, STMN1, HCD2, ALDOC, NFM, IPYR, NDUS1, MDHC, DDAH1, PSD13, APOA1, 6PGL, PEBP1, TTHY, ACTY, IDH3A (**Figure 4D**).

In order to improve the discriminating power of the identified protein markers, we went on to intersect PCA and PLS-DA loading plots and find the shared proteins/best discriminators for each condition (**Figure 3C**). HBA, HBD and HCD2 positively correlated with high-grade gliomas; GFAP, PHP14, 6PGL, PSD13, PEA15, TPIS, CRYAB_b, IPYR and IDH3A correlated with low-grade tumors; on the other hand, NFM, CN37, NDUS1 and MDHC negatively correlated with tumor samples. APOA1, PRDX3_a and CLIC1 were the best discriminators between tumors and negative controls.

### Validation of proteomic profiles

The proteomic landscape of human gliomas was then validated by protein immunoblotting, quantifying the expression levels of randomly-selected spots. Protein markers (APO-A1, SOD1, PRDXII, LDHB, ALDOC) were randomly selected (∼10%) among the 48 differentially expressed proteins and analyzed. Western blot chemiluminescence images were acquired at sub-saturation levels and quantified with ImageJ. Silver staining density quantified as above and Western blot signals were then subjected to Spearman's correlation analysis. Paired signal analysis supported the accuracy of proteomic profiles: APOA1 (ρ = 0.550, p_value_ = 0.015), SOD1 (ρ = 0.517, p_value_ = 0.025), LDHB (ρ = 0.517, p_value_ = 0.044), PRDXII upper band (ρ = −0.086, p_value_ = 0.387), PRDXII lower band (ρ = 0.030; p_value_ = 0.458), ALDOC (ρ = 0.771, p_value_ = 0.051). **Table S4** presents *in extenso* Western blotting data, densitometry, normalization procedure details, scatter plots and elliptic confidence intervals.

### Pathway analysis

**Figure 5:**
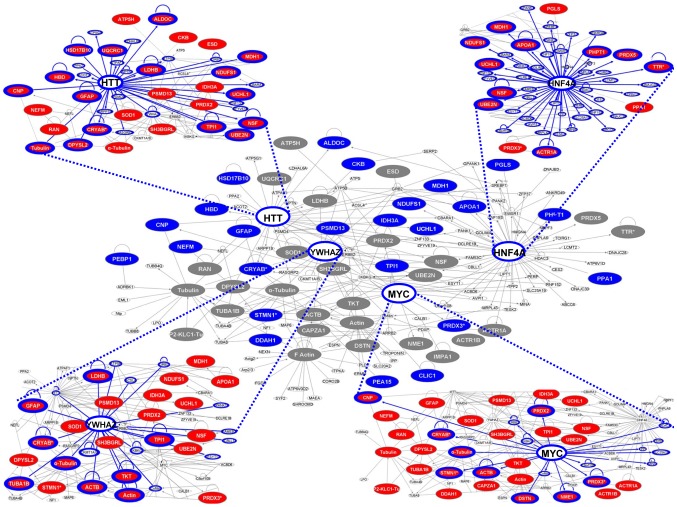
Pathway analysis. Graphical representation of the protein network retrieved using the Ingenuity Pathway Analysis Tool. Proteins are represented as nodes, the biological relationships between the nodes are represented as lines. Proteins identified by PCA and PLS-DA analysis and indicated as discriminant among controls, low-grade and high-grade tumors are in highlighted blue. Most proteins differentially expressed in gliomas are closely connected in the network through four major hubs: Huntingtin, HNF4α, 14-3-3ζ (YWHAZ) and c-Myc. Four external edges: differentially expressed proteins identified by MS (red) and direct interactors with the four major hubs (blue bridges and strengthened contours).

The discriminating performance of the protein clusters identified by unsupervised analysis, and the tight correspondence between PCA and PLS-DA clusters suggested deep, intersected biological relevance. Hence, we went on to explore the existence of a “GBM control module” connecting the discriminating protein clusters through physical and functional interactions. In order to reveal these cross-talks we carried out bioinformatic network detection followed by data meta-analysis. The top-score network (Fisher's exact test: p = 1x10^−104^) was found to contain 46 out of the 48 proteins identified by proteomics (**Figure 5** and **Table S5A**). Most members were found to play key roles in neurological diseases (28), genetic disorders (34), skeletal and muscle diseases (22), and cancer (25). The five top score pathways of this network included proteins involved in mitochondrial function (HCD2, PRDX3, NDUS1, PRDX5 and QCR1), pentose phosphate pathway (6PGL, ALDOC, TKT); glycolysis/gluconeogenesis (HCD2, TPIS, LDHB and ALDOC), inositol metabolism (TPIS and ALDOC) and oxidative phosphorylation (NDUS1, ATP5H, QCR1 and IPYR).

Strikingly, most proteins of the identified network were then found to converge on four major hubs: Hungtintin (HTT, 16 edges), Hepatocyte nuclear factor 4α (*HNF4A*, 10 edges), 14-3-3ζ (*YWHAZ*, 9 edges) and c-Myc (*MYC*, 9 edges) (**Figure 5**). Remarkably, major discriminators identified by PCA and PLS-DA analyses were found to interact with Huntingtin (10), HNF4α (5), c-Myc (4) and 14-3-3ζ (3) (**Table S6**).

We then went on to assess the relevance of the GBM control module versus known molecular pathways involved in GBM biogenesis, via IPA and STRING platforms algorithms (**Tables S5B**). To our surprise, the four hubs we had identified were found to converge on both major players of glioma development and progression: epidermal growth factor receptor (EGFR) and p53. Bridging proteins between network hubs and EGFR/p53 were as follows: UCHL1, TPI1 and SH3BGRL to EGFR; ACTB, CRYAB, STMN1, NME1, Tubulin, GFAP, UBE2N, PPA1 and UCHL1 to p53 (**Table S5B**).

Contrary to a wide-held belief, proteins and mRNA levels correlate poorly in most cellular systems [46]–[48], differential protein/mRNA stability playing a major role in this discordant scenario [49], [50]. Nevertheless, identification of transcription factor-driven differential gene expression landscapes provides insight into tumor-driving gene networks [51]. Hence, we went on to identify upstream transcription factors potentially involved in a coordinated regulation of proteins taking part to the GBM control module. Using stringent criteria for the analysis (P values <0.005, interactions ≥5), we discovered that 9 transcription factors (*HTT, MYC, HNF4A, TP53, ESRRA, NFE2L2, PPARGC1A, MYCN, ESR1*) interacted with 33 out of 48 differentially expressed proteins. Importantly, these transcription factors were also found to interact with/regulate expression of 18 of the best discriminators identified by PCA and PLS-DA (**Table S5C**). Of major relevance, all transcription factors identified as central hubs (Huntingtin, c-Myc, HNF4α) of the GBM control module, together with p53, stood-up as major drivers of the expression of the vast majority of the components of the module.

### Validation of hub expression in tumors

**Figure 6:**
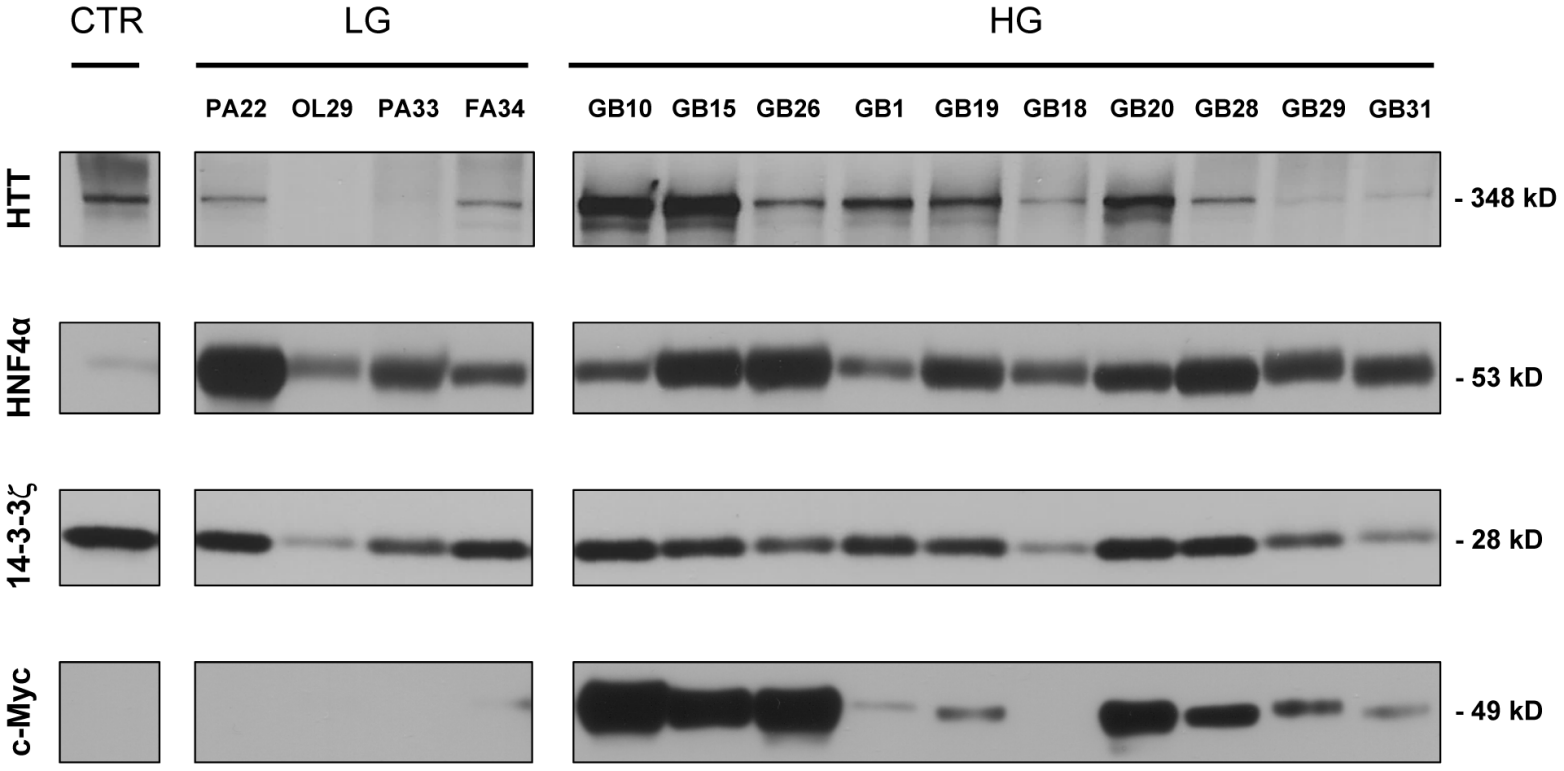
Network hubs expression in glioma samples - Western blot analysis. 14-3-3ζ, HNF4α Huntingtin and c-Myc protein expression levels in tumor and control samples, as determined by Western blotting. GB: glioblastoma mutiforme; OL: oligodendroglioma; PA: pilocytic astrocytoma; FA: fibrillary astrocytoma. Control sample (CTR) was tissue from normal cortex. HG: high-grade tumors. LG: low-grade tumors.

**Figure 7:**
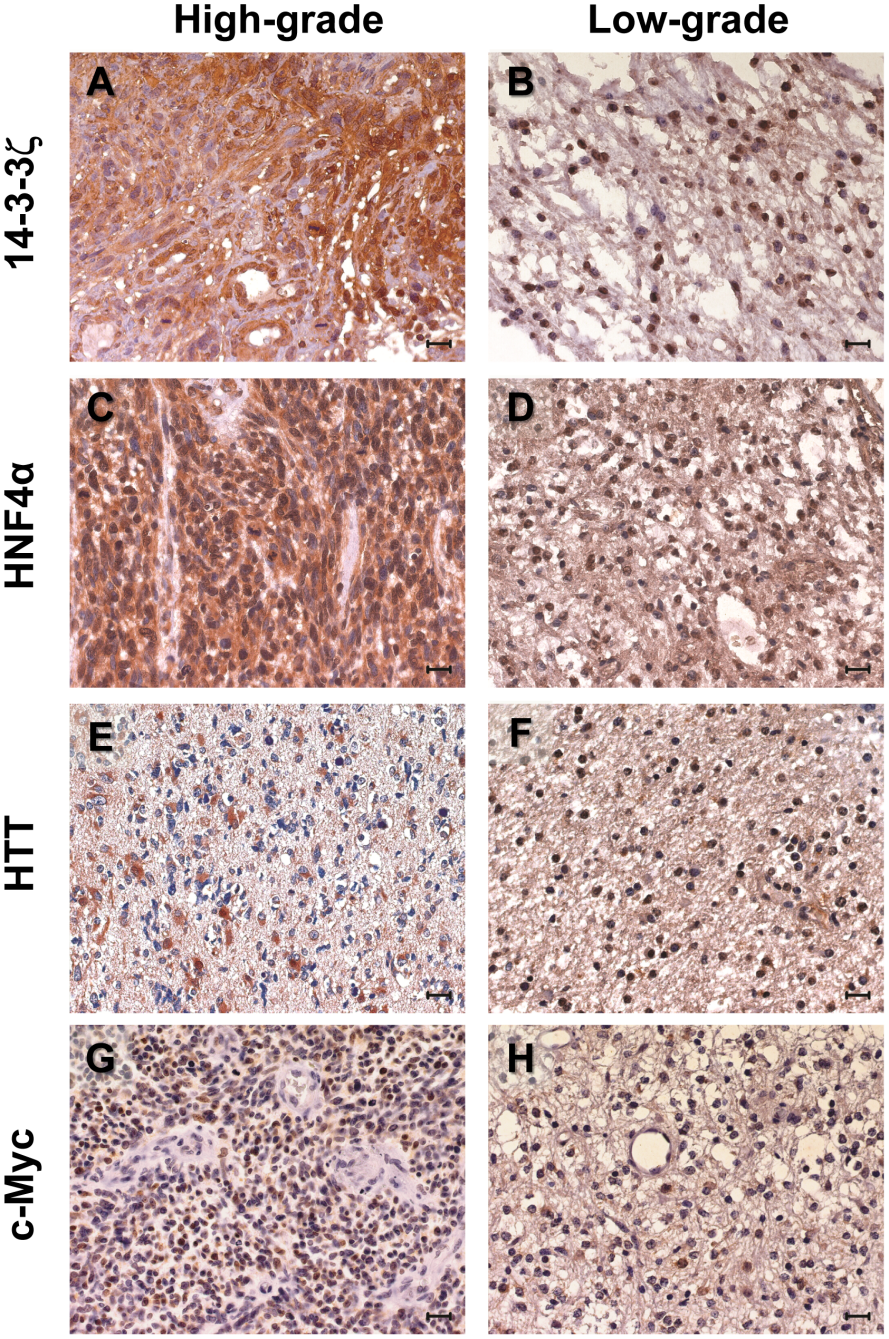
Network hubs expression in glioma samples - IHC analysis. Expression of 14-3-3ζ (A, B), HNF4α (C, D), Huntingtin (E, F) and c-Myc (G, H), as determined by IHC staining of glioblastoma samples (left column) and low-grade tumors (right column). Representative samples are shown. Scale bars  = 20 µm. Nuclei were counterstained with hematoxylin (in blue).

A prediction of our model was that the four hubs of the GBM control module should be broadly expressed. Hence, we assessed their expression in human glioma samples by protein immunoblotting. 14-3-3ζ, HNF4α and Huntingtin were widely expressed in glioma samples. Expression of HNF4α was higher in tumors samples than controls; Huntingtin and c-Myc were found to be overexpressed in high-grade gliomas (**Figure 6**). Moreover, we analyzed the expression of the four hubs in tissue samples by IHC (**Figure 7** and **S5**). In high-grade gliomas 14-3-3ζ and HNF4α were strongly expressed in nuclear and cytoplasmatic compartments (**Figure 7A**, **Figure 7C**). On the other hand, specific nuclear accumulation was observed in low-grade samples (**Figure 7B**, **Figure 7D**). c-Myc was specifically expressed in the nucleus of the glioma samples, with a trend toward an up-regulation from low- to high-grade tumors(**Figure 7G**, **Figure 7H**).

### Four hubs expression meta-analysis

The findings above suggested broad expression of the four hubs of the GBM control module. We verified this prediction by performing a proteome-wide profiling of IHC expression patterns (Human Protein Atlas; www.proteinatlas.org) for Huntingtin, HNF4α, 14-3-3ζ and c-Myc. HNF4α level in normal glial cell showed low levels of staining, with moderate intensity in <25% of the cells. In analyzed gliomas 5/10 had a corresponding expression profile as compared to controls; 2/10 presented an increase of expression (medium staining, moderate intensity and percent reactive cells of 75–25%), 3/10 presented lower expression (<25% of cells) compared with control samples. Hungtintin level in normal glial cell showed low expression (low staining, moderate intensity and percentage <25%) in IHC array stained with mouse mAb. In glioma tissue arrays 0/12 have the same expression profiles compared to controls. Strikingly, 12/12 presented a global increase of expression or a substantial increase of positive cells. A second IHC array set (12 samples) stained with rabbit polyclonal antibody was analyzed confirming this evidence. Consistent with our findings, c-Myc expression in normal glial cells was not detectable by IHC. Rabbit polyclonal antibody targeting the C-terminal portion of c-Myc led to positive staining on 4/11 tumors samples. The mouse mAb gave positive staining in 11/12 astrocytoma samples. 14-3-3ζ presented strong staining levels in normal glial cell (high staining, strong intensity, percentages between 75%–25%). Three out of 10 array samples presented the same staining patterns as controls. The remaining 7/10 samples showed a prevalence of positive cells of >75%. p53 and EGFR expression patterns were analyzed as internal benchmarks of the robustness of analysis and were shown to possess expected expression profiles and prevalence of expression findings (**Table S7**).

## Discussion

Proteomic profiling of human GBM allowed to discover differentially expressed protein clusters, that were shown to craft a tightly interconnected control network. This was recapitulated into a four-hub control module, as centered on Huntingtin, HNF4α, c-Myc and 14-3-3ζ. This was able to stringently discriminate between high-grade GBMs, low-grade tumors and normal tissues. The proteomic clusters included tumor upregulated (PRDX3, APOA1, CLIC1) and downregulated (NFM, NDUS1, MDHC, ALDOC, STMN1, PEBP1, DDAH1, CN37) proteins. Major discriminator between high-grade and low-grade tumors included CRYAB, IPYR, TPIS, PEA15, PSD13, GFAP, IDH3A, 6PGL, PHP14, KCRB as overexpressed in low-grade gliomas; HCD2, HBA, HBD as overexpressed in high-grade GBM. UCHL1 expression showed a positive correlation with normal brain tissue and low-grade tumor, and a negative correlation with high-grade tumors.

Huntingtin, whose mutations are responsible for the neurodegenerative disorders of Huntington's disease, is found in neurites and at synapses, has anti-apoptotic functions and is neuroprotective in brain cells exposed to apoptotic stimuli, such as serum deprivation, mitochondrial toxins or death-inducing genes [52]. Notably, pathogenic Huntingtin affects the expression, redox state, disulfide bonding of antioxidant proteins identified here, among them SODC, and PRDX2, together with PRDXI [53], thus supporting a shared functional link. Taken together, our findings provide first evidence of function of Huntingtin in brain tumors, thus paving novel avenues of investigation on GBM pathophysiology.

HNF4α is a modulator of cell proliferation [54]–[56] through the cell cycle inhibitor p21 [57] and the transmembrane glycoprotein Trop-2 [51]. A tight interplay/feedback loop occurs between HNF4α and c-Myc. Both HNF4α and c-Myc proteins compete for control of the *P21/CDKN1A* gene transcription [57], and deletion of *HNF4A* in hepatocellular carcinoma cells results in significant up-regulation of c-Myc and enhanced cell proliferation rates [58]. Essentially no evidence for expression and function of HNF4α in brain tumors was available before this study, again opening novel avenues for investigation on GBM pathophysiology.

Deregulation of *MYC* is a frequent driver of cancer [59]. c-Myc has been reported to bind a large number of genes [60] and regulates cell proliferation by affecting cell-cycle checkpoint genes, CDK inhibitors and cyclins [61]. c-Myc also plays a major role in regulating metabolic genes required for energy production [62], [63] and ribosomal biogenesis. mTORC2 controls glycolytic metabolism by regulating c-Myc cellular levels and ultimately determines overall survival of GBM patients [64]. This evidence is consistent with our GO analysis showing that major targets of our analysis, such as ALDOC, TPIS, IPYR, 6PGL, HCD2, IDH3A, NDUS1, MDHC are involved in metabolism (e.g. glycolysis, inositol metabolism and oxidative phosphorylation) and cancer-related metabolic reprogramming, including the Warburg effect [65]–[69].

Our findings support a major involvement of 14-3-3ζ in the progression of GBM [70], in agreement with previous studies showing that 14-3-3ζ expression levels were a prognostic factor in GBM [71]. 14-3-3ζ is involved in oral squamous cell [72], stomach [73], breast [74] and papillomavirus-induced carcinomas [75]. Major targets identified in our analysis, such as GFAP, CRYAB and TPIS, are major interactors of 14-3-3ζ and are powerful discriminators of low-grade astrocytoma.

Glioma development is frequently associated with mutations of the isocitrate dehydrogenase *IDH1* and *IDH2* genes [76], [77], whereas mutations of *IDH3* have never been observed in GBM [78]. Our analysis discovered IDH3A quantitative variations in low-grade samples versus high-grade tumors. This provided support for a novel model of interference of IDH proteins in GBM progression, through differential expression of a wild-type protein. Of interest, IDH3A was found to be a specific target for p53-dependent phosphorylation [79], further supporting the functional relevance of the GBM control module.

Remarkably, three of the four hubs of the GBM control module (Huntingtin, HNF4α, c-Myc) are transcription factors. Transcription factor network analysis then highlighted all three of them as major regulators of the expression of most proteins of the GBM control module, supporting a joint driving role in GBM development. A functional role of the newly discovered four-hub control module in GBM appearance and progression further required vast, coordinate expression in tumor cases.

Strikingly, analysis of the transcription factors steering the GBM control module led to the discovery that these tightly interrelate with p53. Notably, p53 can regulate Huntingtin's expression at the transcriptional level [80], thus suggesting a cooperation of these signaling pathways not only in neurological diseases, but also in development and progression of brain tumors. p53 plays a critical role as modulator of the HNF4α/c-Myc feedback loop, since it binds c-Myc [81] as well as HNF4α [82], and inhibits the activity of HNF4α via recruitment of histone deacetylase [82]. Additionally, ATM-dependent activation of p53 involves dephosphorylation and association with 14-3-3 [83].

GBM type II are linked to mutations of *TP53*, whereas GBM type I are thought to be driven by *EGFR* amplification/disregulation [5], [7]. Notably, overexpression of Huntingtin interacting protein 1 (HIP1) has been shown to correlate with increased EGFR levels [84]. HIP1 physically associates with EGFR and maintains its levels in brain tumors [84]. It was recently reported that EGFR induces expression of the oncogenic miRNA miR-7 through a Ras/ERK/Myc pathway, and that c-Myc binds to the miR-7 promoter, enhancing its activity [85]. The 14-3-3ζ protein was reported to directly bind EGFR upon stimulation with EGF [86]. Recently, downregulation of PEPB1/RKIP (Raf kinase inhibitor protein) was shown to be associated with poor outcome and malignant progression [87]. PEPB1 inhibits RTKs signaling blocking Raf/MEK/ERK cascade. We found PEPB1/RKIP downregulated in low-grade and high-grade tumors, extending previous indications [20]. Taken together, our findings indicate deep intertwining of the GBM control module also with EGFR signaling pathway.

In summary, using multitiered proteomic profiling, we discovered previously unidentified hubs (centered on Huntingtin, HNF4α, c-Myc and 14-3-3ζ; these then attract p53 and EGFR) as major signaling drivers in the pathogenesis of brain tumors. Our findings thus support the unexpected existence of a unique GBM control module, helping providing a much needed unifying model for GBM appearance and progression. Future studies should be undertaken to validate a diagnostic/prognostic role of the GBM control module. This may also provide better tools for classification and clinical evaluation of GBM, for more effective procedures for tumor diagnosis, prognosis and patients cure.

## Supporting Information

Figure S1 **Signal versus noise in spot detection.** (A) The gel images were subjected to automatic spot detection setting the same parameters: the number of detected spots was increased in the gray adjusted image (right) as compared with the original one (left). (B) Data normalization view. Box plots and kernel density plots show the distribution of protein concentration before (left) and after (right) autoscaling (mean-centered and divided by the standard deviation of each variable) as described in the text.
(TIF)

Figure S2 **Protein Identification by mass spectrometry.** (A) Red circles indicate spots excised on preparative gels and subjected to in-gel tryptic digestion, followed by MS and MS/MS spectrometry analysis for protein identification. Gels and samples were processed as described in materials and methods section. (B) Examples of mass spectra from identified proteins. Numbers on X axis represent precise m/z values of detected peptide ion signals. The peak masses were used to identify the proteins. For each protein spot the strongest peaks were analyzed by MS/MS fragmentation in LIFT mode. α-cyano-4-hydroxycinnamic acid was used as matrix. (top) P02511, Alpha-crystallin B chain (CRYAB). (middle) P50213 Isocitrate dehydrogenase NAD subunit alpha, mitochondrial (IDH3A). (bottom) P10768, S-formylglutathione hydrolase (ESTD).
(TIF)

Figure S3 **Differential spot expression analysis.** (A) Differentially expressed proteins spots as quantified by image analysis (Materials and Methods) (B–D) Examples of and proteins with multimodal distribution in glioblastomas, low-grade astrocytomas and control samples; protein with higher (APOA1, NFM) or lower discriminating power (TTHY) are shown.
(TIF)

Figures S4 **PLS-DA model permutation test plots and coefficient scores of proteins from the PLS-DA analysis.** Permutation tests for: High-grade tumors (left), low-grade tumors (middle) and controls (right). Permutation tests were performed by comparing goodness of fit and prediction (R^2^ and Q^2^ values) of the original model with the goodness of fit and prediction of several models based on data in which the order of the Y observations were randomly permuted. The two intercepts can be considered as measures of degrees of overfit and overprediction. The correlation coefficients of original and permuted data are reported on the x axis; 200 random permutations were carried out. The values of R^2^ and Q^2^ are reported on the y axis. The two circles on the in the upper right (ρ = 1) correspond to the values of R^2^ (green circles) and Q^2^ (blue circles) of the original data. The other circles represent permutation results. The low values of intercepts show that the model has a statistical significance (not over-fitting). (B) Coefficient scores were utilized to provide an estimate of the protein changes in the various groups. Larger coefficient scores (positive or negative) indicate stronger correlations with proteomic group profile classification. The highest positive (black boxes) or negative (red boxes) discriminating coefficient scores of high-grade tumors (left) were exemplified by translation to low-grade tumors (middle) and controls (right).
(TIF)

Figure S5 **IHC staining in positive control samples.** Expression of the 4-hub proteins in positive control tissue sections. (A) Expression of Huntingtin in small bowel. (B) Expression of HNF4α in small bowel. (C) Expression of 14-3-3ζ in breast cancer. (D) Expression of c-Myc in colon cancer. Scale bars  = 20 µm. Nuclei were counterstained with hematoxylin (in blue).
(TIF)

Table S1 **Inter-gel spot volume quantification variance analysis.** Three replica gels of a reference protein lysate from high-grade brain tumor were utilized for inter-gel variance analysis. Ten randomly chosen spot volumes were quantified using Image Master Platinum 6.0; basic/automated procedure was compared with operator-guided contour drawing. The bar graph shows the gain-of-signal (expressed as percent of variation) detected using operator-guided contour drawing versus basic/automated analysis. P = 0.037 (comparison among means in manual vs basic/automated procedures). SD, standard deviation. ^a^: values from basic-automated quantification procedure. ^b^: values from operator-guided quantification procedure. ^c^: values from operator-guided quantification procedure have been normalized on values from basic-automated procedure, and expressed as percent of variation.
(XLSX)

Table S2 Table S2A: Differentially expressed proteins in tumor samples versus normal brain. 2D-gels (5 Control samples, 10 Low-Grade and 25 High-Grade tumors) were processed and quantified as described. Density values from differentially-expressed protein spots were determined. Density normalization on local landmarking was performed as described in the main text. Table S2B: Protein level distributions in normal brain cortex and tumor samples. Box and scatter plots of protein markers defined by proteomics analysis. The graphs show normalized density values. The boxes encompass values from the first quartile (bottom) to the third quartile (top) for the three category (CTR = control; LG = Low-Grade; HG = Hi-Grade). Red horizontal line, median value. Red cross, average value. Each black dot represents an individual sample.
(XLSX)

Table S3 **Spearman correlation matrix.** Spearman's correlation matrix of all marker proteins identified by MS analysis. Numeric values of Spearman's correlation coefficients (ρ) between variables are reported. Each column and row show individual variables. Global correlation analyses are presented in Figure 3.
(XLSX)

Table S4 **Validation of proteomic target proteins by immunoblotting analysis.** Immunoblot analysis (second column) versus silver normalized density values (first column). Five proteins (∼10%) were randomly selected among the 48 differentially expressed proteins and analyzed in tumor samples. Density values from blots were quantified as described in Materials and Methods, as normalized on red Ponceau signal. Silver staining density and Western blot signals were subjected to Spearman's correlation analysis; correlation coefficients (rho) and p-values are reported. Scatter plots for the two variables with confidence ellipses were generated. Representative blots from APOA1, SOD1, LDHB, PRDXII (upper and lower bands) ALDOC are shown.
(XLSX)

Table S5 Table S5A: Ingenuity Pathway Analysis. The significance values for canonical pathways and other biological functions were calculated using the right-tailed Fisher's exact test by comparing the number of user-specified proteins that participate in a given function or pathway, relative to the total number of occurrences of these proteins in all pathway or functional annotations stored in the Ingenuity pathway knowledge base (IPKB). ^a^: The degree of interaction between differentially expressed markers was compared with that expected by chance. A p-value  = 1×10^−104^ was computed by a hypergeometric test. Table S5B: Supervised pathway analysis. Interaction of EGFR (A) and p53 (B) with network proteins, as determined by IPA analysis. (C) Pathway analysis, as performed by STRING 9.1, of the four major hubs (HTT, HNF4A, Myc, YWHAZ) cross-interacting with p53 and EGFR. Table S5C: Transcription factor pathway analysis. Transcription Factor Analysis, as performed by IPA Upstream Regulator Analysis Tool. Using stringent cut-offs for interaction significance (p value <0.005); a threshold value for interaction with ≥5 target proteins was applied. Nine transcription factors (HTT, MYC, HNF4A, TP53, ESRRA, NFE2L2, PPARGC1A, MYCN, ESR1) were shown to modulate 33 out of 48 differentially expressed proteins. Color codes correspond to those of discriminating proteins by PCA and PLS-DA analysis (Figure 4). Proteins that positively correlate with controls are in red; with low-grade tumors are in blue, with both high-grade and low-grade are in magenta. Correlation of UCHL1 with low-grade/control group is in yellow.
(XLSX)

Table S6 Table S6A: Discriminator proteins from PCA/PLS-DA clusters mapping on network hubs - HTT. Table S6B: Discriminator proteins from PCA/PLS-DA clusters mapping on network hubs - HNF4A. Table S6C: Discriminator proteins from PCA/PLS-DA clusters mapping on network hubs - Myc. Table S6D: Discriminator proteins from PCA/PLS-DA clusters mapping on network hubs - YWHAZ (14-3-3 zeta protein). Protein members from the four discriminator clusters, as defined by PCA and PLS-DA analysis, were mapped on the IPA network (highlighted in blue).
(XLSX)

Table S7 Table S7A: Immunohistochemistry proteome profiling meta-analysis - Huntingtin. Representative examples of IHC-stained sections of glioma samples. Specific staining for the huntingtin protein is identifiable as brown spots. Nuclei are counterstained with hematoxylin (in blue). Table S7B: Immunohistochemistry proteome profiling meta-analysi - HNF4A. Representative examples of IHC-stained sections of glioma samples. Specific staining for the HNF4α protein is identifiable as brown spots. Nuclei are counterstained with hematoxylin (in blue). Table S7C: Immunohistochemistry proteome profiling meta-analysis - c-Myc. Representative examples of IHC-stained sections of glioma samples. Specific staining for the c-Myc protein is identifiable as brown spots. Nuclei are counterstained with hematoxylin (in blue). Table S7D: Immunohistochemistry proteome profiling meta-analysis - YWHAZ (14-3-3ζ protein). Representative examples of IHC-stained sections of glioma samples. Specific staining for YWHAZ (14-3-3ζ protein) is identifiable as brown spots. Nuclei are counterstained with hematoxylin (in blue). Table S7E: Immunohistochemistry proteome profiling meta-analysis - EGFR. Representative examples of IHC-stained sections of glioma samples. Specific staining for the EGFR protein is identifiable as brown spots. Nuclei are counterstained with hematoxylin (in blue). Table S7F: Immunohistochemistry proteome profiling meta-analysis - p53. Representative examples of IHC-stained sections of glioma samples. Specific staining for the p53 protein is identifiable as brown spots. Nuclei are counterstained with hematoxylin (in blue).
(XLSX)

Supplemental Material S1 (DOC)
