# Supplementary figures and images for: A Unique Four-Hub Protein Cluster Associates to Glioblastoma Progression
Source: PLoS One. 2014 Jul 22;9(7):e103030. doi: 10.1371/journal.pone.0103030 (PMC4106866; doi:10.1371/journal.pone.0103030)

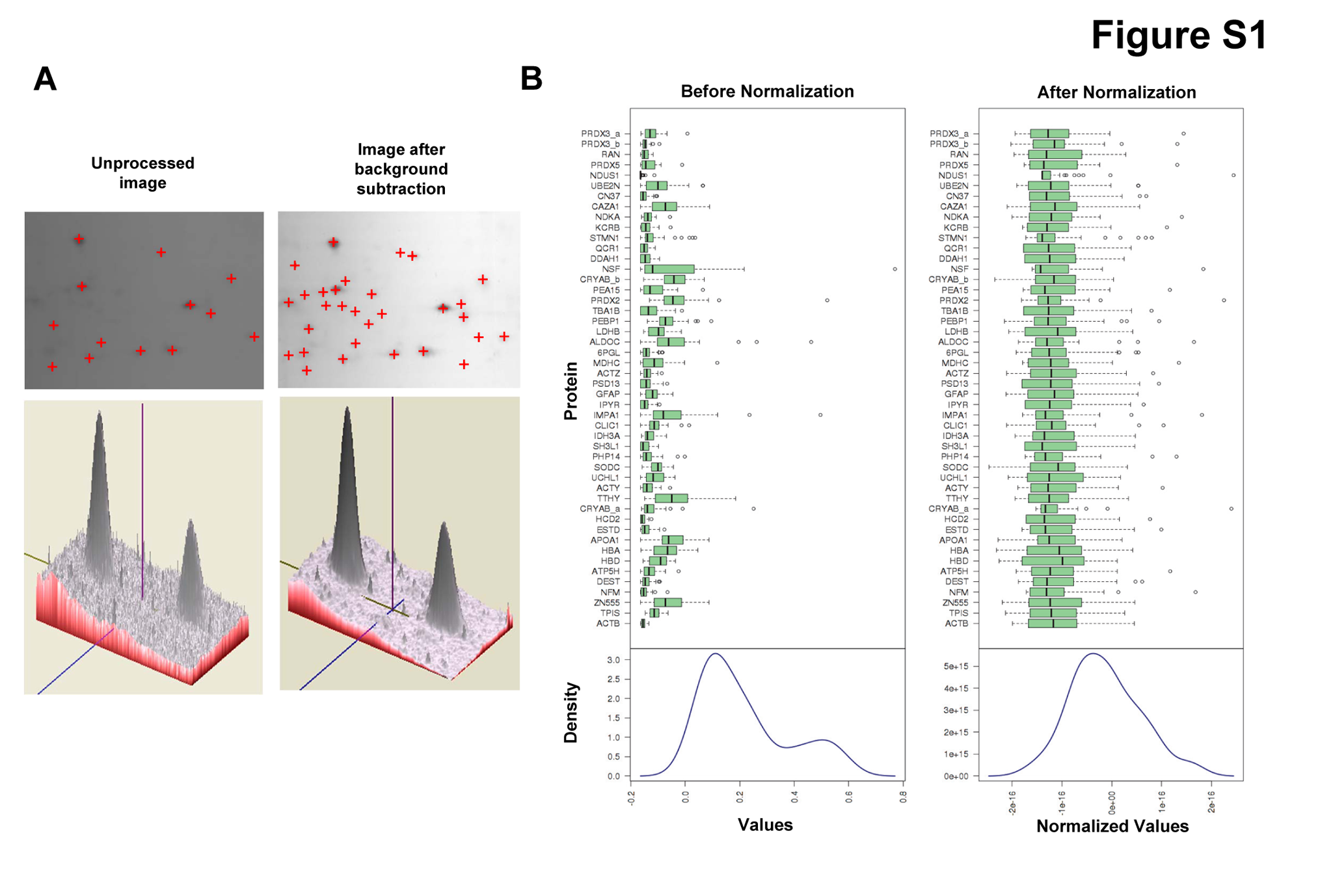

Supplement: Figure S1 — Signal versus noise in spot detection. (A) The gel images were subjected to automatic spot detection setting the same parameters: the number of detected spots was increased in the gray adjusted image (right) as compared with the original one (left). (B) Data normalization view. Box plots and kernel density plots show the distribution of protein concentration before (left) and after (right) autoscaling (mean-centered and divided by the standard deviation of each variable) as described in the text. (TIF) [file pone.0103030.s001.tif]

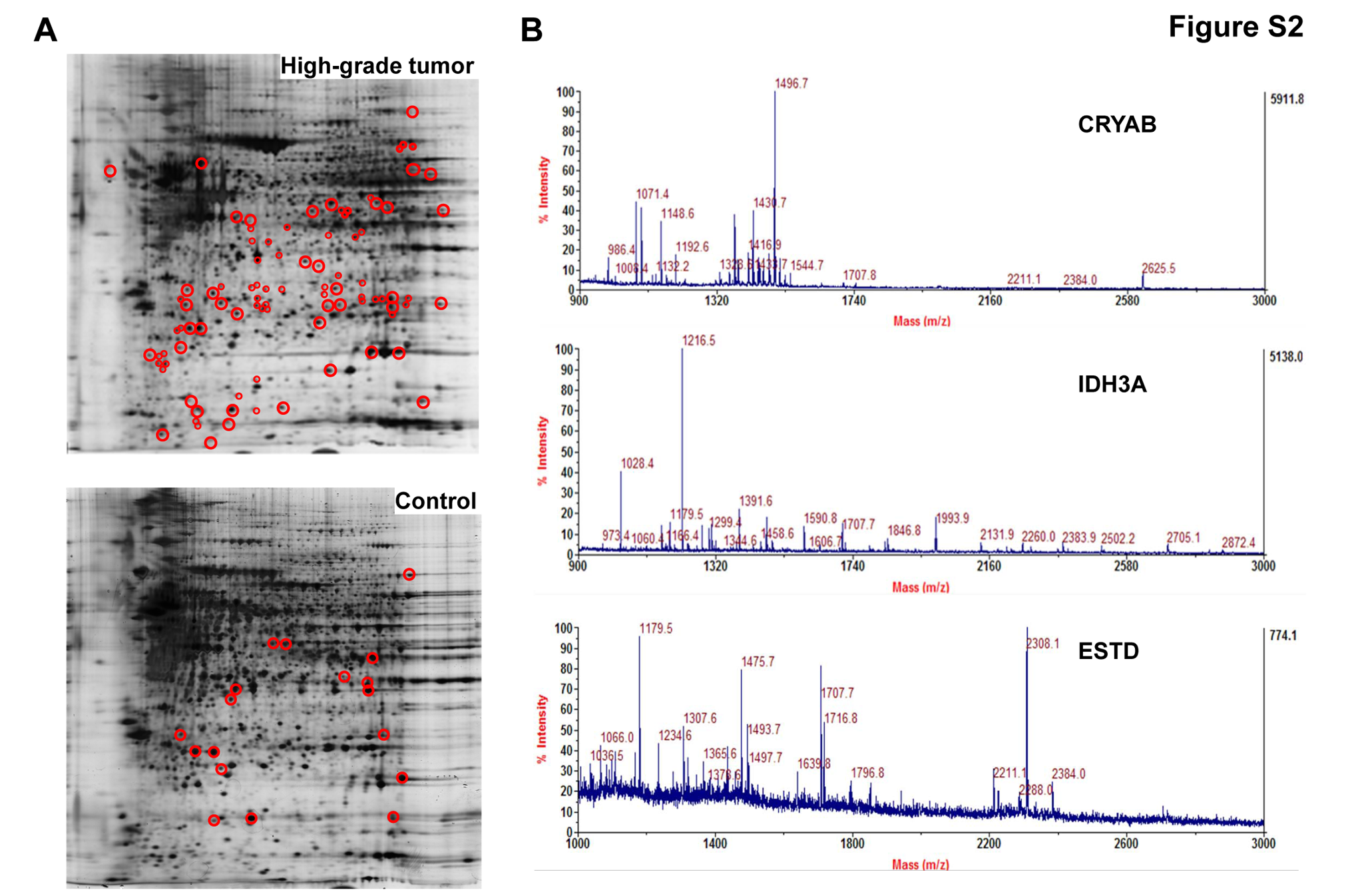

Supplement: Figure S2 — Protein Identification by mass spectrometry. (A) Red circles indicate spots excised on preparative gels and subjected to in-gel tryptic digestion, followed by MS and MS/MS spectrometry analysis for protein identification. Gels and samples were processed as described in materials and methods section. (B) Examples of mass spectra from identified proteins. Numbers on X axis represent precise m/z values of detected peptide ion signals. The peak masses were used to identify the proteins. For each protein spot the strongest peaks were analyzed by MS/MS fragmentation in LIFT mode. α-cyano-4-hydroxycinnamic acid was used as matrix. (top) P02511, Alpha-crystallin B chain (CRYAB). (middle) P50213 Isocitrate dehydrogenase NAD subunit alpha, mitochondrial (IDH3A). (bottom) P10768, S-formylglutathione hydrolase (ESTD). (TIF) [file pone.0103030.s002.tif]

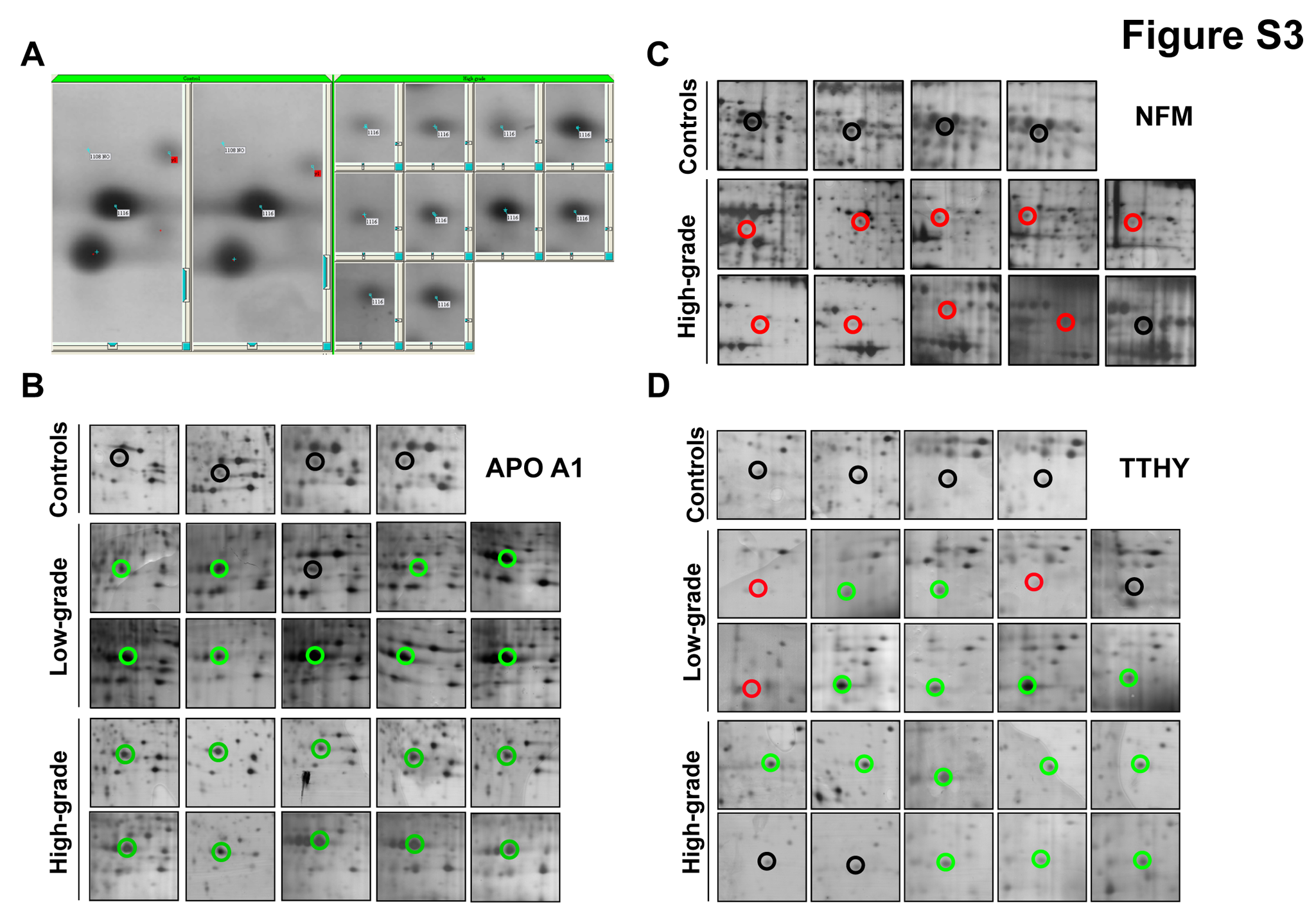

Supplement: Figure S3 — Differential spot expression analysis. (A) Differentially expressed proteins spots as quantified by image analysis (Materials and Methods) (B–D) Examples of and proteins with multimodal distribution in glioblastomas, low-grade astrocytomas and control samples; protein with higher (APOA1, NFM) or lower discriminating power (TTHY) are shown. (TIF) [file pone.0103030.s003.tif]

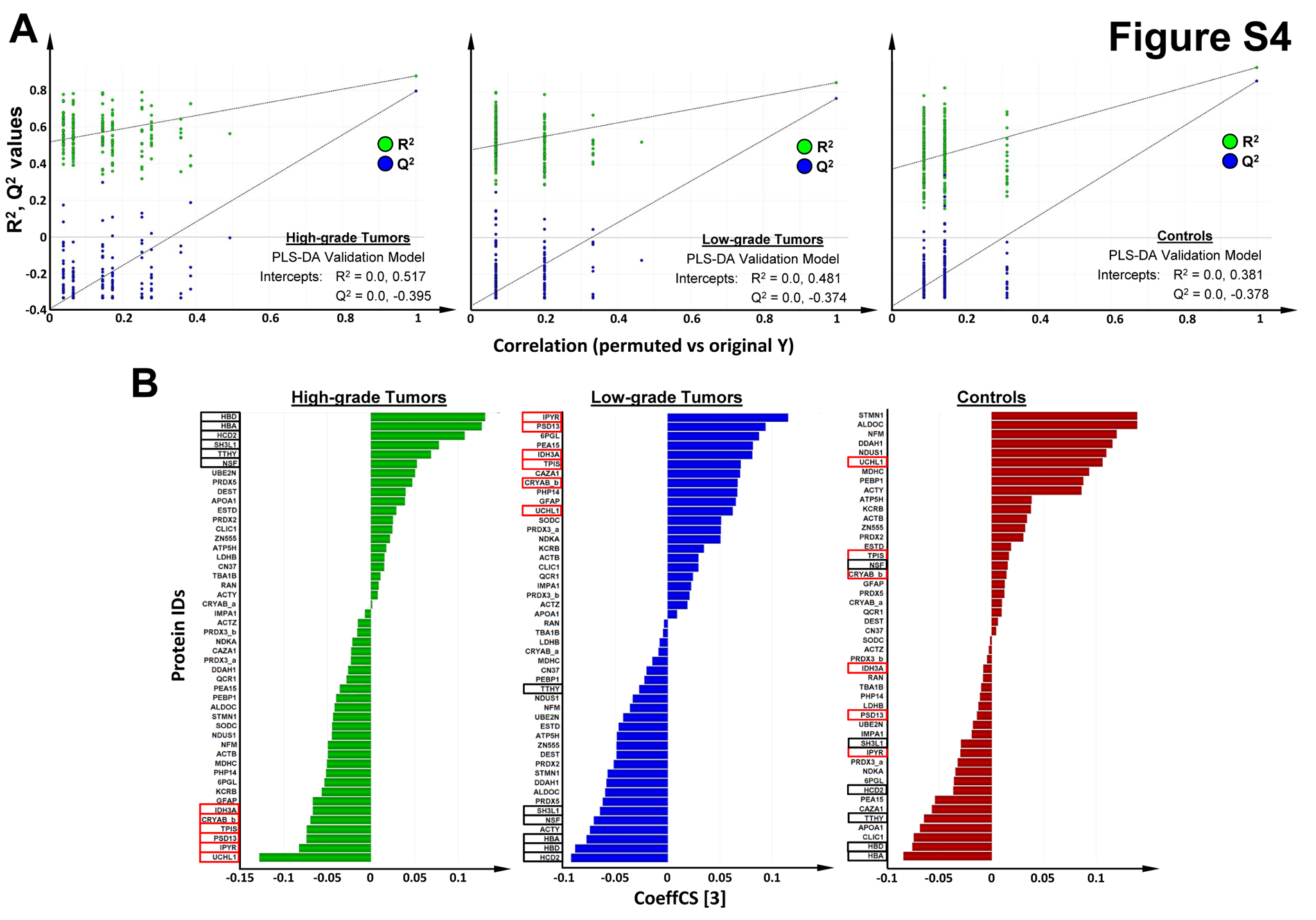

Supplement: Figures S4 — PLS-DA model permutation test plots and coefficient scores of proteins from the PLS-DA analysis. Permutation tests for: High-grade tumors (left), low-grade tumors (middle) and controls (right). Permutation tests were performed by comparing goodness of fit and prediction (R2 and Q2 values) of the original model with the goodness of fit and prediction of several models based on data in which the order of the Y observations were randomly permuted. The two intercepts can be considered as measures of degrees of overfit and overprediction. The correlation coefficients of original and permuted data are reported on the x axis; 200 random permutations were carried out. The values of R2 and Q2 are reported on the y axis. The two circles on the in the upper right (ρ = 1) correspond to the values of R2 (green circles) and Q2 (blue circles) of the original data. The other circles represent permutation results. The low values of intercepts show that the model has a statistical significance (not over-fitting). (B) Coefficient scores were utilized to provide an estimate of the protein changes in the various groups. Larger coefficient scores (positive or negative) indicate stronger correlations with proteomic group profile classification. The highest positive (black boxes) or negative (red boxes) discriminating coefficient scores of high-grade tumors (left) were exemplified by translation to low-grade tumors (middle) and controls (right). (TIF) [file pone.0103030.s004.tif]

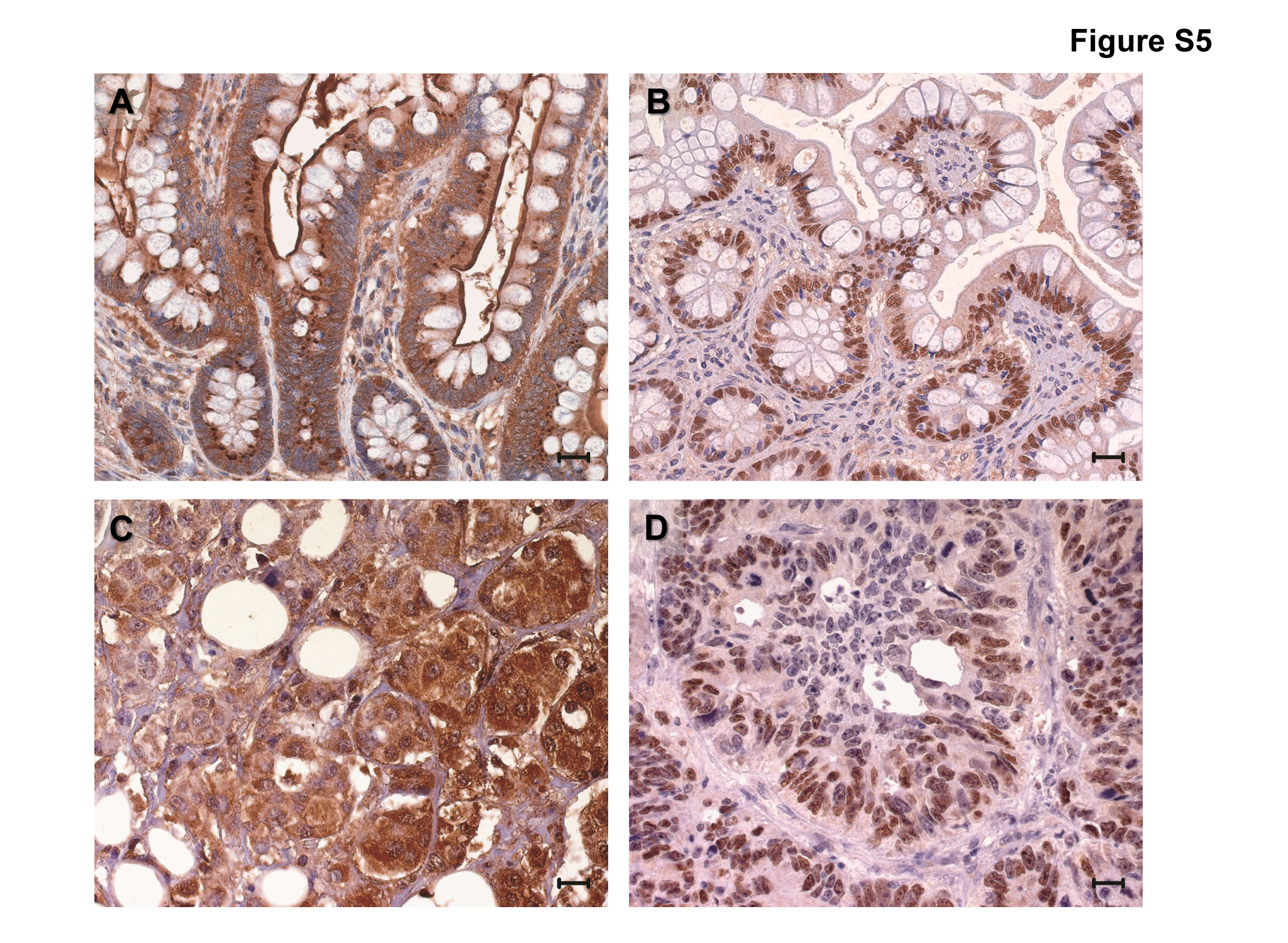

Supplement: Figure S5 — IHC staining in positive control samples. Expression of the 4-hub proteins in positive control tissue sections. (A) Expression of Huntingtin in small bowel. (B) Expression of HNF4α in small bowel. (C) Expression of 14-3-3ζ in breast cancer. (D) Expression of c-Myc in colon cancer. Scale bars = 20 µm. Nuclei were counterstained with hematoxylin (in blue). (TIF) [file pone.0103030.s005.tif]
