# Supplementary material for: A Unique Four-Hub Protein Cluster Associates to Glioblastoma Progression
Source: PLoS One. 2014 Jul 22;9(7):e103030. doi: 10.1371/journal.pone.0103030 (PMC4106866; doi:10.1371/journal.pone.0103030)
Supplement: Supplemental Material S1 — (DOC) [file pone.0103030.s013.doc]

**Supplemental online data**

**Proteins identified by MALDI TOF/TOF.**

(Experimentally determined protein fragments are in red).

**Beta-centractin**

P42025 ACTY_HUMAN

Protein names:

*Recommended name*: **Beta-centractin**

*Alternative name(s)*: **Actin-related protein 1B (Short name: ARP1B**)

Gene names Name: **ACTR1B** Synonyms: **CTRN2**

**1** MESYDIIANQ PVVIDNGSGV IKAGFAGDQI PKYCFPNYVG RPKHMRVMAG

**51** ALEGDLFIGP KAEEHRGLLT IRYPMEHGVV RDWNDMERIW QYVYSKDQLQ

**101** TFSEEHPVLL TEAPLNPSKN REKAAEVFFE TFNVPALFIS MQAVLSLYAT

**151** GRTTGVVLDS GDGVTHAVPI YEGFAMPHSI MRVDIAGRDV SRYLRLLLRK

**201** **EGVDFHTSAE FEVVR**TIKER ACYLSINPQK DEALETEK**VQ YTLPDGSTLD**

**251** **VGPAR**FRAPE LLFQPDLVGD ESEGLHEVVA FAIHKSDMDL RRTLFANIVL

**301** SGGSTLFKGF GDRLLSEVKK LAPKDIKIKI SAPQERLYST WIGGSILASL

**351** DTFKKMWVSK KEYEEDGSRA IHRKTF

**Alpha-crystallin B chain (a)**

P02511 CRYAB_HUMAN

Protein names:

*Recommended name*: **Alpha-crystallin B chain**

*Alternative name*(s): **Alpha(B)-crystallin, Heat shock protein beta-5 (Short name=HspB5);Renal carcinoma antigen NY-REN-27; Rosenthal fiber component**

Gene names Name: **CRYAB** Synonyms: **CRYA2**

**1** MDIAIHHPWI R**RPFFPFHSP** **SR**LFDQFFGE HLLESDLFPT STSLSPFYLR

**51** PPSFLR**APSW** **FDTGLSEMR**L EK**DRFSVNLD** **VKHFSPEELK** VKVLGDVIEV

**101** HGKHEER**QDE HGFISR**EFHR KYRIPADVDP LTITSSLSSD GVLTVNGPRK

**151** QVSGPERTIP ITREEKPAVT AAPKK

**Alpha-crystallin B chain (b)**

P02511 CRYAB_HUMAN

**1** MDIAIHHPWI R**RPFFPFHSP SR**LFDQFFGE HLLESDLFPT STSLSPFYLR

**51** PPSFLR**APSW FDTGLSEMR**L EKDRFSVNLD VKHFSPEELK VKVLGDVIEV

**101** HGKHEERQDE HGFISREFHR KYRIPADVDP LTITSSLSSD GVLTVNGPRK

**151** QVSGPERTIP ITREEKPAVT AAPKK

**Transthyretin**

P02766 TTHY_HUMAN

Protein names:

*Recommended name****:* Transthyretin**

*Alternative name(s)****:*ATTR, Prealbumin, TBPA**

Gene names Name**:TTR** Synonyms**:PALB**

**1** MASHRLLLLC LAGLVFVSEA GPTGTGESKC PLMVKVLDAV R**GSPAINVAV**

**51 HVFR**KAADDT WEPFASGKTS ESGELHGLTT EEEFVEGIYK VEIDTKSYWK

**101** ALGISPFHEH AEVVFTANDS GPRRYTIAAL LSPYSYSTTA VVTNPKE

**3-hydroxyacyl-CoA dehydrogenase type-2**

Q99714 HCD2_HUMAN

Protein names:

*Recommended name*: **3-hydroxyacyl-CoA dehydrogenase type-2**

*Alternative name(s)*: **17-beta-hydroxysteroid dehydrogenase 10 (Short name=17-beta-HSD 10), 3-hydroxy-2-methylbutyryl-CoA dehydrogenase, 3-hydroxyacyl-CoA dehydrogenase type II, Endoplasmic reticulum-associated amyloid beta-peptide-binding protein, Mitochondrial ribonuclease P protein 2 (Short name=Mitochondrial RNase P protein 2), Short-chain type dehydrogenase/reductase XH98G2, Type II HADH**

Gene names Name:**HSD17B10** Synonyms: **ERAB, HADH2, MRPP2, SCHAD, XH98G2**

**1** MAAACRSVK**G LVAVITGGAS GLGLATAER**L VGQGASAVLL DLPNSGGEAQ

**51** AKKLGNNCVF APADVTSEKD VQTALALAKG KFGRVDVAVN CAGIAVASKT

**101** YNLK**KGQTHT LEDFQRVLDV** **NLMGTFNVIR LVAGEMGQNE PDQGGQR**GVI

**151** INTASVAAFE GQVGQAAYSA SK**GGIVGMTL** **PIAR**DLAPIG IRVMTIAPGL

**201** FGTPLLTSLP EK**VCNFLASQ VPFPSR**LGDP AEYAHLVQAI IENPFLNGEV

**251** IRLDGAIRMQ P

**Hemoglobin subunit delta**

P02042 HBD_HUMAN

Protein names:

*Recommended name*: **Hemoglobin subunit delta**

*Alternative name(s)*: **Delta-globin, Hemoglobin delta chain**

Gene names Name:**HBD**

**1** MVHLTPEEKT AVNALWGK**VN VDAVGGEALG RLLVVYPWTQ RFFESFGDLS**

**51 SPDAVMGNPK** VKAHGKK**VLG AFSDGLAHLD NLK**GTFSQLS ELHCDK**LHVD**

**101 PENFRLLGNV LVCVLAR**NFG K**EFTPQMQAA YQK**VVAGVAN ALAHKYH

**ATP synthase subunit d, mitochondrial**

O75947 ATP5H_HUMAN

Protein names:

*Recommended name:* **ATP synthase subunit d, mitochondrial (Short name=ATPase subunit d)**

Gene names Name:**ATP5H** ORF Names: **My032**

**1** MAGRKLALK**T IDWVAFAEII PQNQK**AIASS LK**SWNETLTS** **RLAALPENPP**

**51** **AIDWAYYK**AN VAKAGLVDDF EKKFNALKVP VPEDKYTAQV DAEEKEDVKS

**101** CAEWVSLSKA RIVEYEKEME KMKNLIPFDQ MTIEDLNEAF PETKLDKK**KY**

**151** **PYWPHQPIEN L**

**Destrin**

P60981 DEST_HUMAN

Protein names:

*Recommended name:* **Destrin**

*Alternative name(s):* **Actin-depolymerizing factor (Short name=ADF)**

Gene names Name: **DSTN** Synonyms: **ACTDP, DSN**

**1** MASGVQVADE VCRIFYDMKV RKCSTPEEIK KRKKAVIFCL SADKKCIIVE

**51** EGKEILVGDV GVTITDPFKH FVGMLPEKDC R**YALYDASFE TK**ESRKEELM

**101** FFLWAPELAP LKSKMIYASS KDAIKKKFQG IKHECQANGP EDLNRACIAE

**151** KLGGSLIVAF EGCPV

**Isocitrate dehydrogenase [NAD] subunit alpha, mitochondrial**

P50213 IDH3A_HUMAN

Protein names:

*Recommended name:* **Isocitrate dehydrogenase [NAD] subunit alpha, mitochondrial**

*Alternative name(s):* **Isocitric dehydrogenase subunit alpha, NAD(+)-specific ICDH subunit alpha**

Gene names Name: **IDH3A**

**1** MAGPAWISKV SRLLGAFHNP KQVTRGFTGG VQTVTLIPGD GIGPEISAAV

**51** MKIFDAAK**AP IQWEER**NVTA IQGPGGKWMI PSEAKESMDK NKMGLKGPLK

**101** TPIAAGHPSM NLLLRKTFDL YANVRPCVSI EGYK**TPYTDV NIVTIR**ENTE

**151** GEYSGIEHVI VDGVVQSIKL ITEGASKR**IA EFAFEYAR**NN HRSNVTAVHK

**201** ANIMRMSDGL FLQKCREVAE SCKDIKFNEM YLDTVCLNMV QDPSQFDVLV

**251** MPNLYGDILS DLCAGLIGGL GVTPSGNIGA NGVAIFESVH GTAPDIAGKD

**301** MANPTALLLS AVMMLRHMGL FDHAARIEAA CFATIKDGKS LTKDLGGNAK

**351** **CSDFTEEICR** RVKDLD

**Nucleoside diphosphate kinase A**

P15531 NDKA_HUMAN

Protein names:

*Recommended name:* **Nucleoside diphosphate kinase A (Short name=NDK A,NDP kinase A)**

*Alternative name(s):* **Granzyme A-activated DNase (Short name=GAAD), Metastasis inhibition factor nm23, Tumor metastatic process-associated protein, nm23-H1**

Gene names Name:**NME1** Synonyms:**NDPKA, NM23**

**1** MANCER**TFIA IKPDGVQR**GL VGEIIKRFEQ KGFRLVGLKF MQASEDLLKE

**51** HYVDLK**DRPF FAGLVKYMHS** **GPVVAMVWEG LNVVK**TGR**VM LGETNPADSK**

**101** **PGTIRGDFCI QVGR**NIIHGS DSVESAEKEI GLWFHPEELV DYTSCAQNWI

**151** YE

**Triosephosphate isomerase**

P60174 TPIS_HUMAN

Protein names:

*Recommended name:* **Triosephosphate isomerase 1 (Short name=TIM)**

*Alternative name(s):***Triose-phosphate isomerase**

Gene names Name: **TPI1** Synonyms: **TPI**

**1** MAEDGEEAEF HFAALYISGQ WPRLRADTDL QRLGSSAMAP SR**KFFVGGNW**

**51** **K**MNGRKQSLG ELIGTLNAAK **VPADTEVVCA PPTAYIDFAR** QKLDPKIAVA

**101** AQNCYKVTNG AFTGEISPGM IK**DCGATWVV LGHSERRHVF GESDELIGQK**

**151** **VAHALAEGLG VIACIGEK**LD EREAGITEKV VFEQTKVIAD NVKDWSK**VVL**

**201** **AYEPVWAIGT GK**TATPQQAQ EVHEKLRGWL KSNVSDAVAQ STRIIYGGSV

**251** TGATCKELAS QPDVDGFLVG GASLKPEFVD IINAKQ

**Dihydropyrimidinase-related protein 2**

Q16555 DPYL2_HUMAN

Protein names

*Recommended name:* **Dihydropyrimidinase-related protein 2 (Short name=DRP-2)**

*Alternative name(s):* **Collapsin response mediator protein 2 (Short name=CRMP-2), N2A3, Unc-33-like phosphoprotein 2 (Short name=ULIP-2)**

Gene names Name: **DPYSL2** Synonyms: **CRMP2, ULIP2**

**1** MSYQGKKNIP RITSDRLLIK GGKIVNDDQS FYADIYMEDG LIKQIGENLI

**51** VPGGVKTIEA HSR**MVIPGGI DVHTR**FQMPD QGMTSADDFF QGTKAALAGG

**101** TTMIIDHVVP EPGTSLLAAF DQWREWADSK SCCDYSLHVD ISEWHKGIQE

**151** EMEALVKDHG VNSFLVYMAF KDR**FQLTDCQ IYEVLSVIR**D IGAIAQVHAE

**201** NGDIIAEEQQ R**ILDLGITGP** **EGHVLSRPEE VEAEAVNR**AI TIANQTNCPL

**251** YITKVMSKSS AEVIAQARKK GTVVYGEPIT ASLGTDGSHY WSKNWAKAAA

**301** FVTSPPLSPD PTTPDFLNSL LSCGDLQVTG SAHCTFNTAQ KAVGKDNFTL

**351** IPEGTNGTEE RMSVIWDKAV VTGKMDENQF VAVTSTNAAK VFNLYPRKGR

**401** IAVGSDADLV IWDPDSVKTI SAK**THNSSLE YNIFEGMECR** GSPLVVISQG

**451** K**IVLEDGTLH VTEGSGR**YIP R**KPFPDFVYK** RIKARSRLAE LRGVPR**GLYD**

**501** **GPVCEVSVTP K**TVTPASSAK TSPAKQQAPP VRNLHQSGFS LSGAQIDDNI

**551** PRRTTQRIVA PPGGRANITS LG

**Apolipoprotein A-I**

P02647 APOA1_HUMAN

Protein names

*Recommended name:* **Apolipoprotein A-I Short name=Apo-AI, ApoA-I**

*Alternative name(s):* **Apolipoprotein A1, Cleaved into the following chain: Apolipoprotein A-I(1-242)**

Gene names Name: **APOA1**

**1** MKAAVLTLAV LFLTGSQARH FWQQDEPPQS PWDRVKDLAT VYVDVLKDSG

**51** RDYVSQFEGS ALGKQLNLKL LDNWDSVTST FSK**LREQLGP VTQEFWDNLE**

**101 KETEGLRQEM SK**DLEEVKAK VQPYLDDFQK **KWQEEMELYR** QKVEPLRAEL

**151** QEGARQKLHE LQEK**LSPLGE EMR**DRARAHV DALR**THLAPY SDELR**QRLAA

**201** RLEALKENGG ARLAEYHAKA TEHLSTLSEK AKPALEDLRQ GLLPVLESFK

**251 VSFLSALEEY TK**KLNTQ

**Zinc finger protein 555**

Q8NEP9 ZN555_HUMAN

Protein names:

*Recommended name:* **Zinc finger protein 555**

Gene names Name: **ZNF555**

**1** MDSVVFEDVA VDFTLEEWAL LDSAQRDLYR DVMLETFQNL ASVDDETQFK

**51** ASGSVSQQDI YGEKIPKESK IATFTRNVSW ASVLGKIWDS LSIEDQTTNQ

**101** GRNLSRNHGL ERLCESNDQC GEALSQIPHL NLYKKIPPGV KQYEYNTYGK

**151** VFMHRRTSLK SPITVHTGHK PYQCQECGQA YSCRSHLRMH VR**THNGERPY**

**201 VCK**LCGKTFP RTSSLNRHVR IHTAEKTYEC KQCGK**AFIDF** **SSLTSHLRSH**

**251 TGEKPYKCK**E CGKAFSYSST FRRHTITHTG EKPYKCKECA EAFSYSSTFR

**301** RHMISHTGEK PHKCKECGEA FSYSSAFRRH MITHTGEKPY ECKQCGKTFI

**351** YLQSFRRHER IHTGEKPYEC KQCGKTFIYP QSFRRHER**TH GGEKPYECNQ**

**401 CGK**AFSHPSS FRGHMRVHTG EKPYECKQCG KTFNWPISLR KHMRTHTREK

**451** PYECKQCGKA FSLSACFR**EH VRMHPEDK**SY ECKLCGKAFY CHISLQKHMR

**501** RHTAEKLYKC KQCGKAFSWP ELLQQHVRTH TVEKPYECKE CGKVFKWPSS

**551** LPIHMRLHTG EKPYQCKHCG KAFNCSSSLR RHVRIHTTEK QYK**CNVGHPP**

**601 ANEFMCSASE K**SHQERDLIK VVNMVLPL

**Ubiquitin carboxyl-terminal hydrolase isozyme L1**

P09936 UCHL1_HUMAN

Protein names

*Recommended name*: **Ubiquitin carboxyl-terminal hydrolase isozyme L1 (Short name=UCH-L1)**

*Alternative name(s):* **Neuron cytoplasmic protein 9.5,PGP 9.5 (Short name=PGP9.5), Ubiquitin thioesterase L1**

Gene names Name: **UCHL1**

**1** MQLKPMEINP EMLNKVLSRL GVAGQWRFVD VLGLEEESLG SVPAPACALL

**51** LLFPLTAQHE NFRKKQIEEL KGQEVSPKVY FMKQTIGNSC GTIGLIHAVA

**101** NNQDKLGFED GSVLKQFLSE TEKMSPEDRA KCFEK**NEAIQ AAHDAVAQEG**

**151 QCR**VDDKVNF HFILFNNVDG HLYELDGRMP FPVNHGASSE DTLLKDAAKV

**201** CREFTEREQG EVRFSAVALC KAA

**Hemoglobin subunit alpha**

P69905 HBA_HUMAN

Protein names

*Recommended name:* **Hemoglobin subunit alpha**

*Alternative name(s):* **Alpha-globin, Hemoglobin alpha chain**

Gene names Name**: HBA1 AND HBA2**

**1** MVLSPADKTN VKAAWGK**VGA HAGEYGAEAL ER|MFLSFPTT KTYFPHFDLS**

**51 HGSAQVK**GHG KKVADALTNA VAHVDDMPNA LSALSDLHAH KLRVDPVNFK

**101** LLSHCLLVTL AAHLPAEFTP AVHASLDKFL ASVSTVLTSK YR

**NADH-ubiquinone oxidoreductase 75 kDa subunit, mitochondrial**

P28331 NDUS1_HUMAN

Protein names

*Recommended name:* **NADH-ubiquinone oxidoreductase 75 kDa subunit, mitochondrial**

*Alternative name(s):* **Complex I-75kD (Short name=CI-75kd)**

Gene names Name: **NDUFS1**

**1** MLRIPVRKAL VGLSKSPKGC VRTTATAASN LIEVFVDGQS VMVEPGTTVL

**51** QACEK**VGMQI** **PRFCYHER**LS VAGNCRMCLV EIEKAPKVVA ACAMPVMKGW

**101** NILTNSEKSK KAREGVMEFL LANHPLDCPI CDQGGECDLQ DQSMMFGNDR

**151** SRFLEGKRAV EDKNIGPLVK TIMTRCIQCT RCIRFASEIA GVDDLGTTGR

**201** GNDMQVGTYI EKMFMSELSG NIIDICPVGA LTSKPYAFTA RPWETRKTES

**251** IDVMDAVGSN IVVSTRTGEV MRILPRMHED INEEWISDKT RFAYDGLKRQ

**301** RLTEPMVRNE K**GLLTYTSWE DALSR**VAGML QSFQGKDVAA IAGGLVDAEA

**351** LVALKDLLNR VDSDTLCTEE VFPTAGAGTD LRSNYLLNTT IAGVEEADVV

**401** LLVGTNPR**FE APLFNAR**IRK SWLHNDLKVA LIGSPVDLTY TYDHLGDSPK

**451** ILQDIASGSH PFSQVLKEAK KPMVVLGSSA LQRNDGAAIL AAVSSIAQKI

**501** RMTSGVTGDW KVMNILHR**IA SQVAALDLGY KPGVEAIR**KN PPK**VLFLLGA**

**551 DGGCITR**QDL PKDCFIIYQG HHGDVGAPIA DVILPGAAYT EKSATYVNTE

**601** GRAQQTKVAV TPPGLAREDW KIIR**ALSEIA GMTLPYDTLD QVR**NRLEEVS

**651** PNLVRYDDIE GANYFQQANE LSKLVNQQLL ADPLVPPQLT IKDFYMTDSI

**701** SRASQTMAKC VKAVTEGAQA VEEPSIC

**S-formylglutathione hydrolase**

P10768 ESTD_HUMAN

Protein names

*Recommended name:* **S-formylglutathione hydrolase (Short name=FGH)**

*Alternative name(s):* **Esterase D, Methylumbelliferyl-acetate deacetylase**

Gene names Name: **ESD**

**1** MALKQISSNK CFGGLQKVFE HDSVELNCKM KFAVYLPPKA ETGKCPALYW

**51** LSGLTCTEQN FISK**SGYHQS ASEHGLVVIA PDTSPR**GCNI KGEDESWDFG

**101** TGAGFYVDAT EDPWKTNYRM YSYVTEELPQ LINANFPVDP QRMSIFGHSM

**151** GGHGALICAL KNPGKYKSVS AFAPICNPVL CPWGKKAFSG YLGTDQSKWK

**201** AYDATHLVKS YPGSQLDILI DQGKDDQFLL DGQLLPDNFI AACTEKKIPV

**251** VFRLQEGYDH SYYFIATFIT DHIRHHAKYL NA

**Actin, cytoplasmic 1**

P60709 ACTB_HUMAN

Protein names:

*Recommended name***: Actin, cytoplasmic 1**

*Alternative name(s):* **Beta-actin**

Gene names Name: **ACTB**

**1** MDDDIAA**LVV DNGSGMCK**AG FAGDDAPR**AV FPSIVGRPR**H QGVMVGMGQK

**51 DSYVGDEAQS K**RGILTLKYP IEHGIVTNWD DMEK**IWHHTF YNELR**VAPEE

**101** HPVLLTEAPL NPKANREKMT QIMFETFNTP AMYVAIQAVL SLYASGRTTG

**151** IVMDSGDGVT HTVPIYEGYA LPHAILRLDL AGRDLTDYLM KILTERGYSF

**201** TTTAEREIVR DIKEKLCYVA LDFEQEMATA ASSSSLEK**SY** **ELPDGQVITI**

**251 GNER**FRCPEA LFQPSFLGME SCGIHETTFN SIMKCDVDIR KDLYANTVLS

**301** GGTTMYPGIA DRMQKEITAL APSTMKIKII APPERKYSVW IGGSILASLS

**351** TFQQMWISK**Q EYDESGPSIV HR**KCF

**Neurofilament medium polipeptide**

P07197 NFM_HUMAN

Protein names

*Recommended name:* **neurofilament medium polypeptide (Short name=NF-M)**

*Alternative name(s):* **160 kDa neurofilament protein, Neurofilament 3,Neurofilament triplet M protein**

Gene names Name: **NEFM** Synonyms: **NEF3, NFM**

**1 MSYTLDSLGN PSAYR**RVTET RSSFSRVSGS PSSGFRSQSW SRGSPSTVSS

**51** SYKRSMLAPR LAYSSAMLSS AESSLDFSQS SSLLNGGSGP GGDYKLSRSN

**101** EKEQLQGLND RFAGYIEKVH YLEQQNKEIE AEIQALRQK**Q ASHAQLGDAY**

**151 DQEIRELRAT LEMVNHEKAQ VQLDSDHLEE DIHR**LKERFE EEARLRDDTE

**201** AAIRALRKDI EEASLVKVEL DKK**VQSLQDE VAFLRSNHEE EVADLLAQIQ**

**251 ASHITVER**KD YLKTDISTAL KEIRSQLESH SDQNMHQAEE WFKCRYAKLT

**301** EAAEQNKEAI RSAKEEIAEY RRQLQSKSIE LESVRGTKES LERQLSDIEE

**351** RHNHDLSSYQ DTIQQLENEL RGTKWEMARH LREYQDLLNV K**MALDIEIAA**

**401 YR**KLLEGEET R**FSTFAGSIT GPLYTHRPPI TISSK**IQKPK VEAPKLKVQH

**451** KFVEEIIEET KVEDEK**SEME EALTAITEEL AVSMKEEK**KE AAEEKEEEPE

**501** AEEEEVAAKK SPVKATAPEV KEEEGEKEEE EGQEEEEEED EGAKSDQAEE

**551** GGSEKEGSSE KEEGEQEEGE TEAEAEGEEA EAKEEKKVEE KSEEVATKEE

**601** LVADAKVEKP EKAKSPVPKS PVEEKGKSPV PKSPVEEKGK SPVPKSPVEE

**651** KGKSPVPKSP VEEKGKSPVS KSPVEEKAKS PVPKSPVEEA KSKAEVGKGE

**701** QKEEEEKEVK EAPKEEKVEK KEEKPKDVPE KKKAESPVKE EAVAEVVTIT

**751** KSVKVHLEKE TKEEGKPLQQ EKEKEK**AGGE GGSEEEGSDK** GAKGSRKEDI

**801** AVNGEVEGKE EVEQETKEKG SGREEEKGVV TNGLDLSPAD EKKGGDKSEE

**851** KVVVTKTVEK ITSEGGDGAT KYITKSVTVT QKVEEHEETF EEKLVSTKKV

**901** EKVTSHAIVK EVTQSD

**Peroxiredoxin-5, mitochondrial**

P30044-2 PRDX5_HUMAN

Protein names

*Recommended name:* **Peroxiredoxin-5, mitochondrial**

*Alternative name(s):* **Alu corepressor 1, Antioxidant enzyme b166 (Short name=AOEB166), Liver tissue 2D-page spot 71B, PLP, Peroxiredoxin V (Short name=Prx-V), Peroxisomal antioxidant enzyme, TPx type VI, Thioredoxin peroxidase PMP20, Thioredoxin reductase**

Gene names Name:**PRDX5** Synonyms: **ACR1** ORF names: **SBBI10**

**1** MAPIK**VGDAI PAVEVFEGEP GNK**VNLAELF KGKKGVLFGV PGAFTPGCSK

**51** **THLPGFVEQA EALK**AK**GVQV VACLSVNDAF VTGEWGR**AHK AEGKVRLLAD

**101** PTGAFGK**ETD LLLDDSLVSI FGNR**RLKRFS MVVQDGIVKA LNVEPDGTGL

**151** TCSLAPNIIS QL

**GTP-binding nuclear protein Ran**

P62826 RAN_HUMAN

Protein names:

*Recommended name:* **GTP-binding nuclear protein Ran**

*Alternative name(s):* **Androgen receptor-associated protein 24, GTPase Ran, Ras-like protein TC4,Ras-related nuclear protein**

Gene names Name: **RAN** Synonyms: **ARA24** ORF Names: **OK/SW-cl.81**

**1** MAAQGEPQVQ FKLVLVGDGG TGKTTFVKRH LTGEFEKKYV ATLGVEVHPL

**51** VFHTNRGPIK FNVWDTAGQE KFGGLRDGYY IQAQCAIIMF DVTSRVTYKN

**101** VPNWHRDLVR VCENIPIVLC GNKVDIKDRK VKAKSIVFHR KKNLQYYDIS

**151** AK**SNYNFEKP FLWLAR**KLIG DPNLEFVAMP ALAPPEVVMD PALAAQYEHD

**201** LEVAQTTALP DEDDDL

**Peroxiredoxin-3 (a)**

P30048 PRDX3_HUMAN

Protein names:

*Recommended name*: **Thioredoxin-dependent peroxide reductase, mitochondrial**

*Alternative name(s)*: **Antioxidant protein 1 (Short name=AOP-1), HBC189, Peroxiredoxin III (Short name=Prx-III), Peroxiredoxin-3, Protein MER5 homolog**

Gene names Name: **PRDX3**  Synonyms: **AOP1**

**1** MAAAVGRLLR ASVARHVSAI PWGISATAAL RPAACGRTSL TNLLCSGSSQ

**51** AKLFSTSSSC HAPAVTQHAP YFKGTAVVNG EFKDLSLDDF KGKYLVLFFY

**101** PLDFTFVCPT EIVAFSDKAN EFHDVNCEVV AVSVDSHFSH LAWINTPRKN

**151** GGLGHMNIAL LSDLTKQISR **DYGVLLEGSG LALR**GLFIID PNGVIK**HLSV**

**201 NDLPVGR**SVE ETLRLVKAFQ YVETHGEVCP ANWTPDSPTI KPSPAASKEY

**251** FQKVNQ

**Peroxiredoxin-3 (b)**

**1** MAAAVGRLLR ASVARHVSAI PWGISATAAL RPAACGRTSL TNLLCSGSSQ

**51** AKLFSTSSSC HAPAVTQHAP YFKGTAVVNG EFKDLSLDDF KGKYLVLFFY

**101** PLDFTFVCPT EIVAFSDKAN EFHDVNCEVV AVSVDSHFSH LAWINTPRKN

**151** GGLGHMNIAL LSDLTKQISR **DYGVLLEGSG LALR**GLFIID PNGVIK**HLSV**

**201 NDLPVGR**SVE ETLRLVKAFQ YVETHGEVCP ANWTPDSPTI KPSPAASKEY

**251** FQKVNQ

**Ubiquitin-conjugated enzyme E2 N**

P61088 UBE2N_HUMAN

Protein names

*Recommended name:* **Ubiquitin-conjugating enzyme E2 N**

*Alternative name(s):* **Bendless-like ubiquitin-conjugating enzyme, Ubc13, UbcH13, Ubiquitin carrier protein N, Ubiquitin-protein ligase N**

Gene names Name: **UBE2N** Synonyms: **BLU**

**1** MAGLPRRIIK ETQR**LLAEPV PGIK**AEPDES NAR**YFHVVIA GPQDSPFEGG**

**51 TFKLELFLPE EYPMAAPK**VR FMTKIYHPNV DKLGRICLDI LK**DKWSPALQ**

**101 IR**TVLLSIQA LLSAPNPDDP LANDVAEQWK TNEAQAIETA RAWTRLYAMN

**151** NI

**Transketolase**

P29401 TKT_HUMAN

Protein names

*Recommended name:* **Transketolase (Short name=TK)**

Gene names Name: **TKT**

**1** MESYHKPDQQ KLQALKDTAN RLRISSIQAT TAAGSGHPTS CCSAAEIMAV

**51** LFFHTMRYKS QDPRNPHNDR FVLSKGHAAP ILYAVWAEAG FLAEAELLNL

**101** RKISSDLDGH PVPKQAFTDV ATGSLGQGLG AACGMAYTGK YFDKASYRVY

**151** CLLGDGELSE GSVWEAMAFA SIYK**LDNLVA ILDINR**LGQS DPAPLQHQMD

**201** IYQKRCEAFG WHAIIVDGHS VEELCKAFGQ AKHQPTAIIA KTFKGRGITG

**251** VEDKESWHGK PLPKNMAEQI IQEIYSQIQS KKK**ILATPPQ EDAPSVDIAN**

**301 IR**MPSLPSYK VGDKIATRKA YGQALAKLGH ASDRIIALDG DTKNSTFSEI

**351** FKKEHPDR**FI ECYIAEQNMV SIAVGCATR**N RTVPFCSTFA AFFTRAFDQI

**401** RMAAISESNI NLCGSHCGVS IGEDGPSQMA LEDLAMFRSV PTSTVFYPSD

**451** GVATEKAVEL AANTKGICFI RTSRPENAII YNNNEDFQVG QAKVVLKSKD

**501** DQVTVIGAGV TLHEALAAAE LLKKEKINIR **VLDPFTIKPL DR**KLILDSAR

**551** ATKGRILTVE DHYYEGGIGE AVSSAVVGEP GITVTHLAVN RVPRSGKPAE

**601** LLKMFGIDRD AIAQAVRGLI TKA

**Alpha-centractin**

P61163 ACTZ_HUMAN

Protein names

*Recommended name:* **Alpha-centractin (Short name=Centractin)**

*Alternative name(s):* **ARP1, Actin-RPV, Centrosome-associated actin homolog**

Gene names Name:**ACTR1A**  Synonyms: **CTRN1**

**1** MESYDVIANQ PVVIDNGSGV IKAGFAGDQI PKYCFPNYVG RPKHVR**VMAG**

**51 ALEGDIFIGP K**AEEHRGLLS IR**YPMEHGIV K**DWNDMERIW QYVYSKDQLQ

**101** TFSEEHPVLL TEAPLNPRKN RERAAEVFFE TFNVPALFIS MQAVLSLYAT

**151** GRTTGVVLDS GDGVTHAVPI YEGFAMPHSI MRIDIAGRDV SRFLRLYLRK

**201** EGYDFHSSSE FEIVKAIKER ACYLSINPQK DETLETEK**AQ YYLPDGSTIE**

**251 IGPSR**FRAPE LLFRPDLIGE ESEGIHEVLV FAIQKSDMDL RR**TLFSNIVL**

**301 SGGSTLFK**GF GDRLLSEVKK LAPKDVKIRI SAPQERLYST WIGGSILASL

**351** DTFKKMWVSK KEYEEDGARS IHRKTF

**Malate dehydrogenase, cytoplasmic**

P40925 MDHC_HUMAN

Protein names

*Recommended name:* **Malate dehydrogenase, cytoplasmic**

*Alternative name(s):* **Cytosolic malate dehydrogenase, Diiodophenylpyruvate reductase**

Gene names Name: **MDH1** Synonyms: **MDHA**

**1** MSEPIRVLVT GAAGQIAYSL LYSIGNGSVF GKDQPIILVL LDITPMMGVL

**51** DGVLMELQDC ALPLLKDVIA TDKEDVAFK**D LDVAILVGSM PR**REGMERKD

**101** LLKANVKIFK SQGAALDKYA KKSVKVIVVG NPANTNCLTA SKSAPSIPK**E**

**151 NFSCLTR**LDH NRAKAQIALK LGVTANDVKN VIIWGNHSST QYPDVNHAKV

**201** KLQGK**EVGVY EALKDDSWLK** GEFVTTVQQR GAAVIKARKL SSAMSAAKAI

**251** CDHVRDIWFG TPEGEFVSMG VISDGNSYGV PDDLLYSFPV VIKNKTWK**FV**

**301 EGLPINDFSR** EKMDLTAKEL TEEKESAFEF LSSA

**6-phosphogluconolactonase**

O95336 6PGL_HUMAN

Protein names

*Recommended name:* **6-phosphogluconolactonase (Short name=6PGL)**

Gene names Name:**PGLS**

**1** MAAPAPGLIS VFSSSQELGA ALAQLVAQRA ACCLAGARAR **FALGLSGGSL**

**51 VSMLARELPA AVAPAGPASL ARWTLGFCDE RLVPFDHAES TYGLYR**THLL

**101** SRLPIPESQV ITINPELPVE EAAEDYAKKL RQAFQGDSIP VFDLLILGVG

**151** PDGHTCSLFP DHPLLQEREK **IVAPISDSPK PPPQRVTLTL PVLNAAR**TVI

**201** FVATGEGKAA VLKRILEDQE ENPLPAALVQ PHTGK**LCWFL DEAAAR**LLTV

**251** PFEKHSTL

**Fructose-bisphosphate aldolase C**

P09972 ALDOC_HUMAN

Protein names

*Recommended name:* **Fructose-bisphosphate aldolase C**

*Alternative name(s):* **Brain-type aldolase,**

Gene names Name: **ALDOC** Synonyms: **ALDC**

**1** MPHSYPALSA EQKKELSDIA LRIVAPGKGI LAADESVGSM AKR**LSQIGVE**

**51 NTEENR**RLYR **QVLFSADDR**V KKCIGGVIFF HETLYQKDDN GVPFVRTIQD

**101** KGIVVGIKVD K**GVVPLAGTD GETTTQGLDG LSER**CAQYKK DGADFAKWRC

**151** VLKISER**TPS ALAILENANV LAR**YASICQQ NGIVPIVEPE ILPDGDHDLK

**201** RCQYVTEKVL AAVYKALSDH HVYLEGTLLK PNMVTPGHAC PIK**YTPEEIA**

**251 MATVTALR**RT VPPAVPGVTF LSGGQSEEEA SFNLNAINRC PLPRPWALTF

**301** SYGR**ALQASA LNAWR**GQRDN AGAATEEFIK RAEVNGLAAQ GKYEGSGEDG

**351** GAAAQSLYIA NHAY

**L-lactate dehydrogenase B chain**

P07195 LDHB_HUMAN

Protein names

*Recommended name:* **L-lactate dehydrogenase B chain (Short name=LDH-B)**

*Alternative name(s):* **LDH heart subunit (Short name=LDH-H), Renal carcinoma antigen NY-REN-46**

Gene names Name: **LDHB**

**1** MATLKEK**LIA PVAEEEATVP NNK**ITVVGVG QVGMACAISI LGK**SLADELA**

**51 LVDVLEDK**LK **GEMMDLQHGS LFLQTPK**IVA DKDYSVTANS KIVVVTAGVR

**101** QQEGESRLNL VQRNVNVFKF IIPQIVKYSP DCIIIVVSNP VDILTYVTWK

**151** LSGLPKHR**VI GSGCNLDSAR** FRYLMAEKLG IHPSSCHGWI LGEHGDSSVA

**201** VWSGVNVAGV SLQELNPEMG TDNDSENWKE VHK**MVVESAY EVIK**LKGYTN

**251** WAIGLSVADL IESMLKNLSR IHPVSTMVK**G MYGIENEVFL SLPCILNAR**G

**301** LTSVINQKLK DDEVAQLKK**S ADTLWDIQK**D LKDL

**Phosphatidylethanolamine-binding protein 1**

P30086 PEBP1_HUMAN

Protein names

*Recommended name:* **Phosphatidylethanolamine-binding protein 1 (Short name=PEBP-1)**

*Alternative name(s):* **HCNPpp, Neuropolypeptide h3, Prostatic-binding protein, Raf kinase inhibitor protein (Short name=RKIP)** *Cleaved into the following chain*: **Hippocampal cholinergic neurostimulating peptide (Short name=HCNP)**

Gene names Name: **PEBP1** Synonyms:**PBP, PEBP**

**1** MPVDLSKWSG PLSLQEVDEQ PQHPLHVTYA GAAVDELGKV LTPTQVK**NRP**

**51 TSISWDGLDS GKLYTLVLTD PDAPSR**KDPK YREWHHFLVV NMK**GNDISSG**

**101 TVLSDYVGSG PPK**GTGLHR**Y VWLVYEQDRP LK**CDEPILSN RSGDHRGKFK

**151** VASFRKKYEL R**APVAGTCYQ AEWDDYVPK**L YEQLSGK

**Inositol monophosphatase 1**

P29218 IMPA1_HUMAN

Protein names

*Recommended name:* **Inositol monophosphatase 1 (Short name=IMP 1, IMPase 1)**

*Alternative name(s):* **Inositol-1(or 4)-monophosphatase 1, Lithium-sensitive myo-inositol monophosphatase A1**

Gene names Name: **IMPA1**  Synonyms: **IMPA**

**1** MADPWQECMD YAVTLARQAG EVVCEAIKNE MNVMLKSSPV DLVTATDQKV

**51** EKMLISSIKE KYPSHSFIGE ESVAAGEKSI LTDNPTWIID PIDGTTNFVH

**101** RFPFVAVSIG FAVNKKIEFG VVYSCVEGKM YTARKGKGAF CNGQKLQVSQ

**151** QEDITK**SLLV TELGSSR**TPE TVRMVLSNME K**LFCIPVHGI R**SVGTAAVNM

**201** CLVATGGADA YYEMGIHCWD VAGAGIIVTE AGGVLMDVTG GPFDLMSRRV

**251** IAANNRILAE RIAK**EIQVIP LQR**DDED

**Superoxide dismutase [Cu-Zn]**

P00441 SODC_HUMAN

Protein names

*Recommended name:* **Superoxide dismutase [Cu-Zn]**

*Alternative name(s):* **Superoxide dismutase 1** (Short name=hSod1)

Gene names Name: **SOD1**

**1** MATKAVCVLK **GDGPVQGIIN FEQK**ESNGPV KVWGSIKGLT EGLHGFHVHE

**51** FGDNTAGCTS AGPHFNPLSR KHGGPKDEER **HVGDLGNVTA DK**DGVADVSI

**101** EDSVISLSGD HCIIGRTLVV HEKADDLGKG GNEESTKTGN AGSRLACGVI

**151** GIAQ

**Chloride intracellular channel protein 1**

O00299 CLIC1_HUMAN

Protein names

*Recommended name:* **Chloride intracellular channel protein 1**

*Alternative name(s):* **Chloride channel ABP, Nuclear chloride ion channel 27 (Short name=NCC27), Regulatory nuclear chloride ion channel protein (Short name=hRNCC)**

Gene names Name: **CLIC1** Synonyms: **G6, NCC27**

**1** MAEEQPQVEL FVKAGSDGAK **IGNCPFSQR**L FMVLWLKGVT FNVTTVDTKR

**51** RTETVQKLCP GGQLPFLLYG TEVHTDTNK**I EEFLEAVLCP PR**YPKLAALN

**101** PESNTAGLDI FAKFSAYIKN SNPALNDNLE KGLLKALKVL DNYLTSPLPE

**151** EVDETSAEDE GVSQRKFLDG NELTLADCNL LPKLHIVQVV CKKYR**GFTIP**

**201 EAFR**GVHRYL SNAYAREEFA STCPDDEEIE LAYEQVAKAL K

**Glial fibrillary acidic protein**

P14136 GFAP_HUMAN

Protein names

*Recommended name:* **Glial fibrillary acidic protein (Short name=GFAP)**

Gene names Name: **GFAP**

**1** MERRRITSAA RRSYVSSGEM MVGGLAPGRR LGPGTRLSLA RMPPPLPTRV

**51** DFSLAGALNA GFKETRASER AEMMELNDRF ASYIEKVRFL EQQNKALAAE

**101** LNQLRAKEPT KLADVYQAEL RELRLRLDQL TANSARLEVE RDNLAQDLAT

**151** VRQKLQDETN LRLEAENNLA AYRQEADEAT LARLDLERKI ESLEEEIRFL

**201** RKIHEEEVRE LQEQLARQQV HVELDVAKPD LTAALKEIRT QYEAMASSNM

**251** HEAEEWYRSK FADLTDAAAR NAELLRQAKH EANDYRRQLQ SLTCDLESLR

**301** GTNESLERQM REQEERHVRE AASYQEALAR LEEEGQSLKD EMARHLQEYQ

**351** DLLNVK**LALD IEIATYR**KLL EGEENR**ITIP VQTFSNLQIR** ETSLDTKSVS

**401** EGHLKRNIVV KTVEMRDGEV IKESKQEHKD VM

**Inorganic pyrophosphatase**

Q15181 IPYR_HUMAN

Protein names

*Recommended name:* **Inorganic pyrophosphatase**

*Alternative name(s):* **Pyrophosphate phospho-hydrolase (Short name=PPase)**

Gene names Name: **PPA1** Synonyms: **IOPPP, PP**

**1** MSGFSTEERA APFSLEYRVF LKNEKGQYIS PFHDIPIYAD K**DVFHMVVEV**

**51** **PR**WSNAKMEI ATKDPLNPIK QDVKKGKLRY VANLFPYKGY IWNYGAIPQT

**101** WEDPGHNDKH TGCCGDNDPI DVCEIGSKVC ARGEIIGVKV LGILAMIDEG

**151** ETDWKVIAIN VDDPDAANYN DINDVKR**LKP GYLEATVDWF R**RYKVPDGKP

**201** ENEFAFNAEF KDKDFAIDII KSTHDHWKAL VTKKTNGKGI SCMNTTLSES

**251** PFKCDPDAAR AIVDALPPPC ESACTVPTDV DKWFHHQKN

**14 kDa phosphohistidine phosphatase**

Q9NRX4 PHP14_HUMAN

Protein names

*Recommended name:* **14 kDa phosphohistidine phosphatase**

*Alternative name(s):* **Phosphohistidine phosphatase 1, Protein janus-A homolog**

Gene names Name: **PHPT1** Synonyms: **PHP14** ORF Names:**CGI-202, HSPC141**

**1** MAVADLALIP DVDIDSDGVF KYVLIRVHSA PRSGAPAAES KEIVRGYK**WA**

**51 EYHADIYDK**V SGDMQK**QGCD CECLGGGR**IS HQSQDKKIHV YGYSMAYGPA

**101** QHAISTEKIK AKYPDYEVTW ANDGY

**26S proteasome non-ATPase regulatory subunit 13**

Q9UNM6 PSD13_HUMAN

Protein names

*Recommended name:* **26S proteasome non-ATPase regulatory subunit 13**

*Alternative name(s):* **26S proteasome regulatory subunit RPN9**, **26S proteasome regulatory subunit S11, 26S proteasome regulatory subunit p40.5**

Gene names Name: **PSMD13**

**1** MKDVPGFLQQ SQNSGPGQPA VWHRLEELYT KKLWHQLTLQ VLDFVQDPCF

**51** AQGDGLIK**LY ENFISEFEHR|VNPLSLVEII LHVVR**QMTDP NVALTFLEKT

**101** REKVKSSDEA VILCKTAIGA LKLNIGDLQV TKETIEDVEE MLNNLPGVTS

**151** VHSRFYDLSS KYYQTIGNHA SYYKDALRFL GCVDIKDLPV SEQQERAFTL

**201** GLAGLLGEGV FNFGELLMHP VLESLRNTDR QWLIDTLYAF NSGNVERFQT

**251** LKTAWGQQPD LAANEAQLLR KIQLLCLMEM TFTRPANHRQ LTFEEIAKSA

**301** KITVNEVELL VMKALSVGLV KGSIDEVDKR **VHMTWVQPR**V LDLQQIKGMK

**351** DRLEFWCTDV KSMEMLVEHQ AHDILT

**SH3 domain binding glutamic acid-rich like protein**

O75368 SH3L1_HUMAN

Protein names

*Recommended name:* **SH3 domain-binding glutamic acid-rich-like protein**

Gene names Name: **SH3BGRL**

**1** MVIRVYIASS SGSTAIKKKQ QDVLGFLEAN KIGFEEKDIA ANEENRKWMR

**51** ENVPENSRPA TGYPLPPQIF NESQYR**GDYD AFFEAR**ENNA VYAFLGLTAP

**101** PGSKEAEVQA KQQA

**F-actin-capping protein subunit alpha-1**

P52907 CAZA1_HUMAN

Protein names

*Recommended name:* **F-actin-capping protein subunit alpha-1**

*Alternative name(s):* **CapZ alpha-1**

Gene names Name: **CAPZA1**

**1** MADFDDRVSD EEKVRIAAK**F ITHAPPGEFN EVFNDVR**LLL NNDNLLREGA

**51** AHAFAQYNMD QFTPVK**IEGY EDQVLITEHG DLGNSR**FLDP RNKISFKFDH

**101** LRKEASDPQP EEADGGLKSW RESCDSALRA YVKDHYSNGF CTVYAKTIDG

**151** QQTIIACIES HQFQPKNFWN GRWRSEWKFT ITPPTAQVVG VLK**IQVHYYE**

**201 DGNVQLVSHK** DVQDSLTVSN EAQTAKEFIK IIENAENEYQ TAISENYQTM

**251** SDTTFKALRR QLPVTRTKID WNKILSYKIG KEMQNA

**2',3'-cyclic-nucleotide 3'-phosphodiesterase**

P09543 CN37_HUMAN

Protein names

*Recommended name:* **2',3'-cyclic-nucleotide 3'-phosphodiesterase (Short name=CNP, CNPase)**

Gene names Name: **CNP**

**1** MNRGFSRKSH TFLPKIFFRK MSSSGAKDKP ELQFPFLQDE DTVATLLECK

**51** TLFILRGLPG SGKSTLARVI VDKYRDGTKM VSADAYKITP GARGAFSEEY

**101** K**RLDEDLAAY CR**RRDIR**ILV** **LDDTNHER**ER LEQLFEMADQ YQYQVVLVEP

**151** KTAWRLDCAQ LKEKNQWQLS ADDLKKLKPG LEKDFLPLYF GWFLTKKSSE

**201** TLRKAGQVFL EELGNHKAFK KELRQFVPGD EPREKMDLVT YFGKRPPGVL

**251** HCTTKFCDYG KAPGAEEYAQ QDVLKKSYSK AFTLTISALF VTPKTTGARV

**301** ELSEQQLQLW PSDVDKLSPT DNLPRGSRAH ITLGCAADVE AVQTGLDLLE

**351** ILRQEKGGSR GEEVGELSRG KLYSLGNGRW MLTLAKNMEV RAIFTGYYGK

**401** GKPVPTQGSR KGGALQSCTI I

**Peroxiredoxin-2**

P32119 PRDX2_HUMAN

Protein names

*Recommended name:* **Peroxiredoxin-2**

*Alternative name(s):* **Natural killer cell-enhancing factor B (Short name=NKEF-B), PRP, Thiol-specific antioxidant protein (Short name=TSA), Thioredoxin peroxidase 1, Thioredoxin-dependent peroxide reductase 1**

Gene names Name: **PRDX2** Synonyms: **NKEFB, TDPX1**

**1** MASGNARIGK PAPDFKATAV VDGAFKEVKL SDYKGKYVVL FFYPLDFTFV

**51** CPTEIIAFSN RAEDFRKLGC EVLGVSVDSQ FTHLAWINTP R**KEGGLGPLN**

**101** **IPLLADVTR**R LSEDYGVLKT DEGIAYRGLF IIDGKGVLR**Q ITVNDLPVGR**

**151** SVDEALRLVQ AFQYTDEHGE VCPAGWKPGS DTIKPNVDDS KEYFSKHN

**Tubulin alpha-1B chain**

P68363 TBA1B_HUMAN

Protein names

*Recommended name:* **Tubulin alpha-1B chain**

*Alternative name(s):* **Alpha-tubulin ubiquitous, Tubulin K-alpha-1, Tubulin alpha-ubiquitous chain**

Gene names Name: **TUBA1B**

**1**  MRECISIHVG QAGVQIGNAC WELYCLEHGI QPDGQMPSDK TIGGGDDSFN

**51**  TFFSETGAGK HVPR**AVFVDL EPTVIDEVR**T GTYR**QLFHPE QLITGK**EDAA

**101** NNYARGHYTI GKEIIDLVLD RIRKLADQCT GLQGFLVFHS FGGGTGSGFT

**151**  SLLMERLSVD YGKKSKLEFS IYPAPQVSTA VVEPYNSILT THTTLEHSDC

**201**  AFMVDNEAIY DICRRNLDIE RPTYTNLNR**L ISQIVSSITA SLRFDGALNV**

**251** **DLTEFQTNLV PYPRIHFPLA TYAPVISAEK** AYHEQLSVAE ITNACFEPAN

**301** QMVKCDPRHG KYMACCLLYR GDVVPKDVNA AIATIKTKRS IQFVDWCPTG

**351**  FKVGINYQPP TVVPGGDLAK VQRAVCMLSN TTAIAEAWAR LDHKFDLMYA

**401**  KRAFVHWYVG EGMEEGEFSE AREDMAALEK DYEEVGVDSV EGEGEEEGEE

**451** Y

**Astrocytic phosphoprotein PEA-15**

Q15121 PEA15_HUMAN

Protein names

*Recommended name:* **Astrocytic phosphoprotein PEA-15**

*Alternative name(s):* **15 kDa phosphoprotein enriched in astrocytes, Phosphoprotein enriched in diabetes (Short name=PED)**

Gene names Name: **PEA15**

**1** MAEYGTLLQD LTNNITLEDL EQLKSACKED IPSEK**SEEIT TGSAWFSFLE**

**51 SHNKLDKDNL SYIEHIFEIS RRPDLLTMVV DYR**TRVLKIS EEDELDTKLT

**101** RIPSAKKYKD IIRQPSEEEI IKLAPPPKKA

**Vesicle-fusing ATPase**

P46459 NSF_HUMAN

Protein names

Recommended name: **Vesicle-fusing ATPase**

Alternative name(s): **N-ethylmaleimide-sensitive fusion protein (Short name=NEM-sensitive fusion protein), Vesicular-fusion protein NSF**

Gene names Name: **NSF**

**1** MAGRSMQAAR CPTDELSLTN CAVVNEK**DFQ SGQHVIVR**TS PNHRYTFTLK

**51** THPSVVPGSI AFSLPQRKWA GLSIGQEIEV SLYTFDKAKQ CIGTMTIEID

**101** FLQKKSIDSN PYDTDKMAAE FIQQFNNQAF SVGQQLVFSF NEKLFGLLVK

**151** DIEAMDPSIL KGEPATGKRQ KIEVGLVVGN SQVAFEKAEN SSLNLIGKAK

**201** TKENRQSIIN PDWNFEKMGI GGLDKEFSDI FRRAFASRVF PPEIVEQMGC

**251** KHVKGILLYG PPGCGKTLLA RQIGKMLNAR EPKVVNGPEI LNKYVGESEA

**301** NIRKLFADAE EEQRRLGANS GLHIIIFDEI DAICKQRGSM AGSTGVHDTV

**351** VNQLLSKIDG VEQLNNILVI GMTNRPDLID EALLRPGRLE VKMEIGLPDE

**401** KGR**LQILHIH TAR**MRGHQLL SADVDIKELA VETK**NFSGAE LEGLVR**AAQS

**451** TAMNRHIKAS TKVEVDMEKA ESLQVTRGDF LASLENDIKP AFGTNQEDYA

**501** SYIMNGIIKW GDPVTRVLDD GELLVQQTKN SDRTPLVSVL LEGPPHSGKT

**551** ALAAKIAEES NFPFIKICSP DKMIGFSETA KCQAMKKIFD DAYKSQLSCV

**601** VVDDIER**LLD YVPIGPR**FSN LVLQALLVLL KKAPPQGRKL LIIGTTSRKD

**651** VLQEMEMLNA FSTTIHVPNI ATGEQLLEAL ELLGNFKDKE RTTIAQQVKG

**701** KKVWIGIKKL LMLIEMSLQM DPEYRVRKFL ALLREEGASP LDFD

**Dimethylarginine dimethylaminohydrolase 1**

O94760 DDAH1_HUMAN

Protein names

*Recommended name:* **N(G),N(G)-dimethylarginine dimethylaminohydrolase 1( Short name=DDAH-1, Dimethylarginine dimethylaminohydrolase 1)**

*Alternative name(s):* **DDAHI, Dimethylargininase-1**

Gene names Name: **DDAH1** Synonyms: **DDAH**

**1** MAGLGHPAAF GRATHAVVR**A LPESLGQHAL R**SAKGEEVDV ARAER**QHQLY**

**51 VGVLGSK**LGL QVVELPADES LPDCVFVEDV AVVCEETALI TRPGAPSRRK

**101** EVDMMKEALE KLQLNIVEMK **DENATLDGGD VLFTGR**EFFV GLSKRTNQRG

**151** AEILADTFKD YAVSTVPVAD GLHLKSFCSM AGPNLIAIGS SESAQKALKI

**201** MQQMSDHRYD KLTVPDDIAA NCIYLNIPNK GHVLLHRTPE EYPESAKVYE

**251** KLKDHMLIPV SMSELEKVDG LLTCCSVLIN KKVDS

**Cytochrome b-c1 complex subunit 1, mitochondrial**

P31930 QCR1_HUMAN

Protein names

Recommended name: **Cytochrome b-c1 complex subunit 1, mitochondrial**

Alternative name(s): **Complex III subunit 1, Core protein I, Ubiquinol-cytochrome-c reductase complex core protein 1**

Gene names Name: **UQCRC1**

**1** MAASVVCRAA TAGAQVLLRA RRSPALLRTP ALRSTATFAQ ALQFVPETQV

**51** SLLDNGLR**VA SEQSSQPTCT VGVWIDVGSR** FETEKNNGAG YFLEHLAFKG

**101** TKNRPGSALE KEVESMGAHL NAYSTREHTA YYIKALSKDL PKAVELLGDI

**151** VQNCSLEDSQ IEKERDVILR EMQENDASMR DVVFNYLHAT AFQGTPLAQA

**201** VEGPSENVRK LSRADLTEYL STHYKAPRMV LAAAGGVEHQ QLLDLAQK**HL**

**251 GGIPWTYAED AVPTLTPCR**F TGSEIRHRDD ALPFAHVAIA VEGPGWASPD

**301** NVALQVANAI IGHYDCTYGG GVHLSSPLAS GAVANKLCQS FQTFSICYAE

**351** TGLLGAHFVC DRMK**IDDMMF VLQGQWMR**LC TSATESEVAR GKNILR**NALV**

**401 SHLDGTTPVC EDIGR**SLLTY GRRIPLAEWE SRIAEVDASV VREICSKYIY

**451** DQCPAVAGYG PIEQLPDYNR IRSGMFWLRF

**Creatine kinase B-type**

P12277 KCRB_HUMAN

Protein names

*Recommended name:* **Creatine kinase B-type**

*Alternative name(s):* **B-CK, Creatine kinase B chain**

Gene names Name: **CKB**  Synonyms: **CKBB**

**1** MPFSNSHNAL KLRFPAEDEF PDLSAHNNHM AK**VLTPELYA ELR**AKSTPSG

**51** FTLDDVIQTG VDNPGHPYIM TVGCVAGDEE SYEVFKDLFD PIIEDRHGGY

**101** KPSDEHKTDL NPDNLQGGDD LDPNYVLSSR VRTGRSIRGF CLPPHCSRGE

**151** RRAIEK**LAVE ALSSLDGDLA GR**YYALKSMT EAEQQQLIDD HFLFDKPVSP

**201** LLLASGMARD WPDARGIWHN DNK**TFLVWVN EEDHLR**VISM QKGGNMKEVF

**251** TRFCTGLTQI ETLFKSKDYE FMWNPHLGYI LTCPSNLGTG LRAGVHIKLP

**301** NLGKHEKFSE VLKRLRLQKR **GTGGVDTAAV GGVFDVSNAD R**LGFSEVELV

**351** QMVVDGVKLL IEMEQRLEQG QAIDDLMPAQ K

**Stathmin**

P16949 STMN1_HUMAN

Protein names

Recommended name: **Stathmin**

Alternative name(s): **Leukemia-associated phosphoprotein p18, Metablastin, Oncoprotein 18 (Short name=Op18), Phosphoprotein p19 (Short name=pp19), Prosolin, Protein Pr22, pp17**

Gene names Name: **STMN1** Synonyms: **C1orf215, LAP18, OP18**

**1** MASSDIQVKE LEKR**ASGQAF ELILSPR**SK**E SVPEFPLSPP K**KKDLSLEEI

**51** QKKLEAAEER RKSHEAEVLK QLAEKREHEK EVLQKAIEEN NNFSKMAEEK

**101** LTHKMEANKE NREAQMAAKL ERLREKDKHI EEVRKNKESK DPADETEAD
